# TriCLFF: a multi-modal feature fusion framework using contrastive learning for spatial domain identification

**DOI:** 10.1093/bib/bbaf316

**Published:** 2025-07-10

**Authors:** Fenglan Pang, Guangfu Xue, Wenyi Yang, Yideng Cai, Jinhao Que, Haoxiu Sun, Pingping Wang, Shuaiyu Su, Xiyun Jin, Qian Ding, Zuxiang Wang, Meng Luo, Yuexin Yang, Yi Lin, Renjie Tan, Yusong Liu, Zhaochun Xu, Qinghua Jiang

**Affiliations:** Center for Bioinformatics, School of Life Science and Technology, Harbin Institute of Technology, 92 Xidazhi Street, Nangang District, Harbin 150001, Heilongjiang Province, China; Center for Bioinformatics, School of Life Science and Technology, Harbin Institute of Technology, 92 Xidazhi Street, Nangang District, Harbin 150001, Heilongjiang Province, China; Center for Bioinformatics, School of Life Science and Technology, Harbin Institute of Technology, 92 Xidazhi Street, Nangang District, Harbin 150001, Heilongjiang Province, China; Center for Bioinformatics, School of Life Science and Technology, Harbin Institute of Technology, 92 Xidazhi Street, Nangang District, Harbin 150001, Heilongjiang Province, China; Center for Bioinformatics, School of Life Science and Technology, Harbin Institute of Technology, 92 Xidazhi Street, Nangang District, Harbin 150001, Heilongjiang Province, China; School of Interdisciplinary Medicine and Engineering, Harbin Medical University, 157 Baojian Road, Nangang District, Harbin 150081, Heilongjiang Province, China; School of Interdisciplinary Medicine and Engineering, Harbin Medical University, 157 Baojian Road, Nangang District, Harbin 150081, Heilongjiang Province, China; Center for Bioinformatics, School of Life Science and Technology, Harbin Institute of Technology, 92 Xidazhi Street, Nangang District, Harbin 150001, Heilongjiang Province, China; School of Interdisciplinary Medicine and Engineering, Harbin Medical University, 157 Baojian Road, Nangang District, Harbin 150081, Heilongjiang Province, China; Center for Bioinformatics, School of Life Science and Technology, Harbin Institute of Technology, 92 Xidazhi Street, Nangang District, Harbin 150001, Heilongjiang Province, China; School of Interdisciplinary Medicine and Engineering, Harbin Medical University, 157 Baojian Road, Nangang District, Harbin 150081, Heilongjiang Province, China; Center for Bioinformatics, School of Life Science and Technology, Harbin Institute of Technology, 92 Xidazhi Street, Nangang District, Harbin 150001, Heilongjiang Province, China; Center for Bioinformatics, School of Life Science and Technology, Harbin Institute of Technology, 92 Xidazhi Street, Nangang District, Harbin 150001, Heilongjiang Province, China; School of Interdisciplinary Medicine and Engineering, Harbin Medical University, 157 Baojian Road, Nangang District, Harbin 150081, Heilongjiang Province, China; School of Interdisciplinary Medicine and Engineering, Harbin Medical University, 157 Baojian Road, Nangang District, Harbin 150081, Heilongjiang Province, China; School of Interdisciplinary Medicine and Engineering, Harbin Medical University, 157 Baojian Road, Nangang District, Harbin 150081, Heilongjiang Province, China; School of Interdisciplinary Medicine and Engineering, Harbin Medical University, 157 Baojian Road, Nangang District, Harbin 150081, Heilongjiang Province, China; Center for Bioinformatics, School of Life Science and Technology, Harbin Institute of Technology, 92 Xidazhi Street, Nangang District, Harbin 150001, Heilongjiang Province, China; School of Interdisciplinary Medicine and Engineering, Harbin Medical University, 157 Baojian Road, Nangang District, Harbin 150081, Heilongjiang Province, China

**Keywords:** spatial transcriptomics, spatial domain identification, multi-modal learning, contrastive learning, feature fusion

## Abstract

Spatial transcriptomics (ST) encompasses rich multi-modal information related to cell state and organization. Precisely identifying spatial domains with consistent gene expression patterns and histological features is a critical task in ST analysis, which requires comprehensive integration of multi-modal information. Here, we propose TriCLFF, a contrastive learning-based multi-modal feature fusion framework, to effectively integrate spatial associations, gene expression levels, and histological features in a unified manner. Leveraging an advanced feature fusion mechanism, our proposed TriCLFF framework outperforms existing state-of-the-art methods in terms of accuracy and robustness across four datasets (mouse brain anterior, mouse olfactory bulb, human dorsolateral prefrontal cortex, and human breast cancer) from different platforms (10x Visium and Stereo-seq) for spatial domain identification. TriCLFF also facilitates the identification of finer-grained structures in breast cancer tissues and detects previously unknown gene expression patterns in the human dorsolateral prefrontal cortex, providing novel insights for understanding tissue functions. Overall, TriCLFF establishes an effective paradigm for integrating spatial multi-modal data, demonstrating its potential for advancing ST research. The source code of TriCLFF is available online at https://github.com/HBZZ168/TriCLFF.

## Introduction

Spatial transcriptomics (ST) is a broad field that distinguishes itself from other transcriptomic technologies by preserving spatial structural information, enhancing insights into complex biological organizations, and analyzing cellular functions and states [1]. The novel technology has been used to identify spatial heterogeneity within tissues, to study brain disorders [2, 3], tumor microenvironments [4, 5], and embryonic development [6], as well as to identify biomarkers and elucidate potential disease mechanisms [7]. Technologically, ST primarily consists of fluorescence *in situ* hybridization-based multiplex imaging techniques (e.g. seqFISH+ [8] and MERFISH [9]), sequencing-based techniques (e.g. STARmap [10] and 10x Xenium [11]), and *in situ* capture techniques (e.g. Slide-seq [12], 10x Visium [13], and Stereo-seq [14]). However, there are still bottlenecks in widespread applications due to the inadequacy and imperfect tools for ST analysis.

For spatial transcriptomic data, a vital task is to identify regions with similar spatial expression patterns and histological features (i.e. spatial domains), which is essential to reveal complex spatial structures and functions of cells and tissues. This task can be treated as an unsupervised spatial clustering problem in machine learning. While algorithms such as k-means and Louvain have been effective in solving general clustering problems, they perform suboptimal in ST due to their inability to incorporate spatial positional information. Aimed at such a problem, researchers put forward multiple solutions to integrate gene expression profiles and spatial coordinates from both statistical [15, 16] and deep learning [17–19] perspectives. Although these methods have improved the identification accuracy and the continuity of the spatial domains to some extent, their performance has plateaued due to overfitting and potential data quality issues [20, 21].

Given that other data modalities can potentially compensate for the limitations of gene expression profiles, integrating multi-modal features is a promising approach to enhancing spatial domain identification. Histology images can be a reliable predictive modality for identifying spatial domains since such data have been treated as the highest diagnostic basis of complex diseases [22, 23]. StLearn [24] and SpaGCN [25] utilize morphological features of images to enhance the associations between spots. DeepST [26] integrates the extracted image features with gene expression and spatial location to generate a latent representation jointly. Introducing histology images, these methods improved the accuracy of spatial domain identification, revealing that gene expression levels, spatial distribution, and histomorphology information are all vital factors for identifying spatial domains. However, these methods do not sufficiently utilize rich information within multi-modal features, particularly neglecting conflicts among different modalities, which may limit their performance.

Contrastive learning is an approach that focuses on extracting meaningful representations by contrasting positive and negative pairs of instances. It has been proven to be a good solution by exploiting complementary information while avoiding noisy and redundant information within different modalities in multiple research fields [27–32]. Considering that spatial domain identification relies on sparse and noisy transcriptomics data, requires the integration of features from multiple modalities, and demands maintaining spatial consistency among adjacent spots, contrastive learning frameworks emerge as a robust and effective approach for addressing this challenge. Existing studies of ConGI [33] and GraphST [34] have applied contrastive learning in diverse ways for spatial domain identification, but there remains considerable room to enhance the integration of multi-modal features. ConGI integrates gene expression levels with histology images by setting features from the same spot but different modalities as positive pairs. Although this method can effectively align features across different modalities, it may compromise spatial consistency within spatial domains due to the lack of consideration for spatial associations between spots. In contrast, GraphST emphasizes local associations between spots in spatial domain identification, using the gene expression of adjacent spots as positive pairs. However, the absence of supplementary modalities may reduce its robustness and noise resilience. Considering the progress of ConGI and GraphST on identification accuracy, we believe the incorporation of contrastive learning and comprehensive features is a feasible improvement direction for spatial domain identification.

Herein, we propose TriCLFF, a multi-modal feature fusion framework using contrastive learning for spatial domain identification. The framework models three types of features, including association among spots, gene expression levels, and tissue morphological information from gene expression profiles and histology images. Specifically, these three types of features are extracted by graph attention auto-encoder (GATE), multilayer perceptron (MLP), and Swin-Transformer [35], respectively. To capture the complementarity across different types of features, the TriCLFF framework uses contrastive learning, introducing six contrastive loss functions within and between multiple modalities, to efficiently align and fuse features and learn unified and information-rich embeddings of spots. As a result, the learned embeddings are then used to identify the spatial domains via mclust, a Gaussian mixture model (GMM) clustering method [36]. We performed extensive tests and comparisons with existing algorithms on multiple ST datasets [human dorsolateral prefrontal cortex (DLPFC), human breast cancer, mouse brain, and olfactory bulb] generated by different platforms (10x Visium, Stereo-seq) as benchmarks. In addition, TriCLFF facilitates the identification of finer-grained structures in breast cancer and detects previously unknown gene expression patterns in the human DLPFC, providing novel evidence for understanding tissue functions. Taken together, TriCLFF is highly effective in accurately identifying spatial domains and provides novel insights into the structural and functional characteristics of tissues and diseases.

## Material and methods

### Datasets and preprocessing

We evaluate the performance of TriCLFF on datasets from two different platforms (10 X Visium data, Stereo-seq). These datasets cover four different tissues of humans and mice, whose details are illustrated in Supplementary Table S1 [2, 14, 34]. The three 10x Visium datasets (DLPFC, human breast cancer and mouse brain) are provided with manual annotations while the mouse olfactory bulb dataset only has a rough laminar structure.

The datasets we used consist of spot-level gene expression profiles and their location coordinates, as well as corresponding histology images (except for Stereo-seq data). In all datasets, we first removed spots outside the main tissue area. As preprocessing steps, we selected top 3000 highly variable genes and performed log-transformed Count Per Million (CPM) normalization on raw gene expression data using SCANPY package [37]. For histology images, we cropped 112×112-pixel patches at the position of each spot to match the spot diameter, which makes the number of image patches for each slide become consistent to the number of spots.

We employed data augmentation methods for both transcriptomic profiles and image patches. For a given spot, data augmentation techniques such as random swap and random noise are applied to slightly distort the original gene expression ${X}_g$ of the spot, which generates two augmented gene expressions ${\overset{\sim }{X}}_{g\_u}$ and ${\overset{\sim }{X}}_{g\_v}$. Similarly, the original image patch ${X}_i$ is distorted by techniques such as random grayscale, and random cropping to generate two augmented image patches ${\overset{\sim }{X}}_{i\_u}$ and ${\overset{\sim }{X}}_{i\_v}$. Since both augmented inputs are treated in the same way at the encoding process, we simply use ${\overset{\sim }{X}}_g$ and ${\overset{\sim }{X}}_i$ represent any one group of the augmented gene expression and image patches in this paper.

### TriCLFF framework

**Figure 1:**
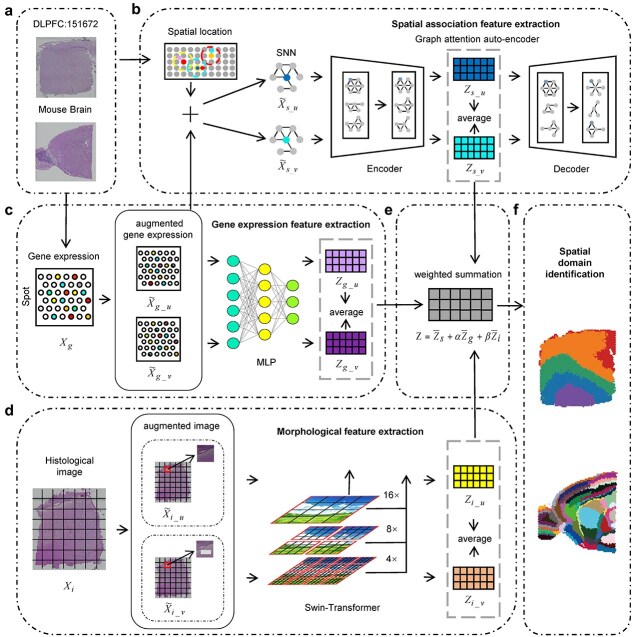
The schematic overview of the TriCLFF framework. The TriCLFF framework takes (a) spatially paired gene expression and image patches as input and generates integrated embeddings as output for each spot. Specifically, the input data are augmented and then fed into three encoders (GATE, MLP encoder, and Swin-Transformer encoder) to extract embeddings for (b) spatial associations, (c) gene expression levels and (d) histological morphology features, respectively. The embedding for each modality is obtained through reconstruction loss and six intra- and inter-modality contrastive learning losses. (e) The final low-dimensional latent embeddings are further integrated using a weighted sum method and subsequently used for (f) spatial domain identification.

TriCLFF is a contrastive learning framework to learn low-dimensional fusion embeddings of gene expression profiles and histology images. As illustrated in Fig. 1, we first utilize three different kinds of encoders (i.e. GATE, MLP and Swin Transformer [35]) to extract embeddings for spatial associations, gene expression levels and histological morphological features individually and then integrate them with a weighted sum method. Since gene expressions at spot-level and spatial-associated level may indicate different biological functions, we treat them as different modalities to enhance the comprehensiveness of feature extraction. To better capture the fusion features, we use contrastive learning strategy in both single-modality and cross-modality levels, which introducing six contrastive learning losses. Combined with GMM clustering method [36], our proposed TriCLFF framework can significantly improve the spatial domain identification accuracy and further benefit to other downstream analyses, such as trajectory inference and Uniform Manifold Approximation and Projection (UMAP) visualization. The implement details of TriCLFF are described in the following sections while the parameters in main analysis pipelines and ablation experiments (except for the parameters are tunning) are provided in Supplementary Table S4.

### Spatial association feature extraction

To model the association among spots, we first use augmented gene expressions ${\overset{\sim }{X}}_g$ and spatial position coordinates to construct spatial neighbor network (SNN) ${\overset{\sim }{X}}_s$ [18] based on Euclidean distance between spots. All spots with available gene expression in a slide are used to construct the network. Spots with spatial distance <$150\ \mathrm{\mu} \mathrm{m}$ are treated as adjacent in this piece of work. We further use GATE [38] to extract spot embeddings. GATE consists of an encoder and a decoder, where the encoder is used to generate a 30-dimensional embedding for each spot and the decoder is used to reconstruct gene expression profiles with the embedding. For both encoder and decoder, the network consists of a two-layer graph attention neural network and a fully connected output layer. For the $k$-th graph attention layer, the output ${h}_i^{(k)}$ of spot $i$ can be expressed by:


(1) \begin{equation*} {h}_i^{(k)}=\mathrm{\sigma} \left({\sum}_{j\in{N}_i}{\alpha}_{ij}^{(k)}\left({W}_k{h}_j^{\left(k-1\right)}\right)\right) \end{equation*}

where ${W}_k$ is the trainable weight matrix, ${N}_i$ denotes the spatial neighborhood of spot $i$ in SNN, ${\alpha}_{ij}^{(k)}$ is attention weight between spot $i$ and $j$ in layer $k$, $\mathrm{\sigma} \left(\cdotp \right)$ is Exponential Linear Unit (ELU) activation function. ${\alpha}_{ij}^{(k)}$ can be represented by:


(2) \begin{equation*} {\displaystyle \begin{array}{c}{\alpha}_{ij}^{(k)}=\frac{\exp \left({e}_{ij}^{(k)}\right)}{\sum_{l\in{N}_i}\exp \left({e}_{il}^{(k)}\right)}\end{array}} \end{equation*}

(3) \begin{equation*} {\displaystyle \begin{array}{c}{e}_{ij}^{(k)}=\mathrm{sigmoid}\left({v}_s^{(k)^T}\mathrm{\sigma} \left({W}_k{h}_i^{\left(k-1\right)}\right)+{v}_r^{(k)^T}\sigma \left({W}_k{h}_j^{\left(k-1\right)}\right)\right)\end{array}} \end{equation*}

where ${v}_s^{(k)}$ and ${v}_r^{(k)}$ are trainable weights for each layer $k$. To avoid overfitting and reduce computational complexity, we directly derive the decoder weights ${\hat{W}}_k$ and ${\hat{\alpha}}_{ij}^{(k)}$ with the corresponding encoder weights ${W}_k$ and ${\alpha}_{ij}^{(k)}$ instead of training (i.e. ${\hat{W}}_k={W}_k^T$ and ${\hat{\alpha}}_{ij}^{(k)}={\alpha}_{ij}^{(k)}$).

### Gene expression feature extraction

We construct a gene expression encoder with MLP to obtain a 30-dimensional embedding for each spot to model gene expression levels. The MLP encoder consists of two LinearBlocks, each containing a fully connected layer, batch normalization layer, ELU activation function, and Dropout layer. Combining all these steps, the transformation formula for the $k$-th LinearBlock can be written as:


(4) \begin{equation*} {\displaystyle \begin{array}{c}{H}^{(k)}=\mathrm{Dropout}\left(\sigma \left(\mathrm{BatchNorm}\left(W\cdotp{H}^{\left(k-1\right)}+b\right)\right),{p}_{drop}\right)\end{array}} \end{equation*}

where $W$ and $b$ are trainable weights, the dropout parameter ${p}_{drop}$ is set to 0.3. This combination of layers is used to ensure that the model can learn complex patterns, maintain stable and efficient training, and avoid overfitting.

### Morphological feature extraction

We employ Swin-Transformer [35], a novel Vision Transformer architecture with pretrained weights, to extract the 30-dimensional morphological feature of each spot from the corresponding image patches. Specifically, we use Swin-S backbone of Swin-Transformer architecture, which includes a patch partition module and four feature extraction stages containing 2, 2, 18, and 2 Swin blocks structures, respectively. Swin-Transformer block is a residual network comprising multi-head self-attention (MSA) or shifted window MSA (SW-MSA) modules, LayerNorm (LN) layers and an MLP output module. The alternating use of MSA and SW-MSA in successive Swin blocks balances computational efficiency and model performance. LN layers are applied before each MSA or SW-MSA module and the MLP module, while residual connections are added after them. We used the default pretrained weights from torchvision for Swin-S backbone and replaced the original classification head with a projection head. The projection head comprises two linear layers, which are connected by a ReLU activation layer. Each linear layer implements an equal-dimensional transformation of 768 dimensions. The output features are compressed to 30 dimensions using a LinearBlock as described in Eq. (4). During this process, the replaced projection head and the feature compression LinearBlock are trainable while the Swin-S backbone is stabilized.

### Loss functions

TriCLFF framework uses reconstruction loss and six intra- and inter-modality contrastive learning losses to learn the trainable weights in an unsupervised manner. Reconstruction loss ${L}_{recon}$ quantifies the difference between the gene expression reconstructed by GATE and the original expression for each spot, which can be formulated by:


(5) \begin{equation*} {\displaystyle{L}_{recon}=\min \sum_{i=1}^N\left\Vert{x}_i-{\hat{x}}_i\right\Vert } \end{equation*}

where ${x}_i$ and ${\hat{x}}_i$ denote original and reconstructed expression of spot $i$, respectively. $N$ is the total number of spots, and $\left\Vert \cdotp \right\Vert$ represents ${\ell}_2$ norm.

We employ identical neural network projection heads for each encoder to map the multi-modal embeddings into a shared latent space, where the contrastive learning loss subsequently calculated. This additional step has been proved to improve the effectiveness of contrastive learning [33]. The projection head is a three-layer fully connected network, with nonlinear activation functions (ReLU or Tanh) between layers. The dimension of projection head outputs is same as the input embeddings. For embeddings ${Z}_m$ of modal $m$, the output of projected embeddings ${\overset{\sim }{Z}}_m$ can be expressed by:


(6) \begin{equation*} {\displaystyle \begin{array}{c}{\overset{\sim }{Z}}_m=\mathrm{Tanh}\left({W}_3\cdotp \mathrm{Relu}\left({W}_2\cdotp \mathrm{Relu}\left({W}_1\cdotp{Z}_m+{b}_1\right)+{b}_2\right)+{b}_3\right)\end{array}} \end{equation*}

where ${W}_1,{W}_2,{W}_3,{b}_1,{b}_2$ and ${b}_3$ are trainable weights.

Contrastive learning losses in TriCLFF are designed following SimCLR [39]. The framework calculates contrastive loss within modality and across modalities in a similar way where the only difference is the pair construction. For each modality, there are paired embeddings derived from different augmentation views. The contrastive loss within modality is computed using paired embeddings in the modality, whereas the cross-modality contrastive loss randomly selects one augmented view in each modality to form paired embeddings for calculation. For each pair of augmented inputs or multi-modal inputs, we randomly select $N$ spots to build mini-batches, which means there are $2N$ embeddings in each mini-batch. Since the embeddings themselves have included the spatial association knowledge, only embeddings on same spots are treated as positive pairs. For each pair of projected embeddings ${\tilde{z}}_i$ and ${\tilde{z}}_j$, their similarity ${l}_{i,j}$ can be evaluated by:


(7) \begin{equation*} {\displaystyle \begin{array}{c}{l}_{i,j}=-\log \frac{\exp \left(s\left({\tilde{z}}_i,{\tilde{z}}_j\right)/\tau \right)}{\sum_{k=1}^{2N}{\mathbb{1}}_{\left[k\ne i\right]}\exp \left(s\left({\tilde{z}}_i,{\tilde{z}}_k\right)/\tau \right)}\end{array}} \end{equation*}

where $\mathrm{s}\left({\tilde{z}}_i,{\tilde{z}}_j\right)={\tilde{z}}_i^T{\tilde{z}}_j{\Big/} \left(\left\Vert{\tilde{z}}_i\right\Vert \cdot \left\Vert{\tilde{z}}_j\right\Vert \right)$, ${\mathbb{1}}_{\left[k\ne i\right]}\in \left\{0,1\right\}$ is an indicator function that equals 1 if the embeddings ${\tilde{z}}_i$ and ${\tilde{z}}_k$ are on different spots, and $\tau$ is the temperature parameter. When constructing similarity matrix ${\left[{l}_{i,j}\right]}_{2N\times 2N}$, the paired embeddings for each spot are adjacent, which means $\forall k\in \left\{1,\dots, N\right\}$, the embeddings whose indices are 2k-1 and 2k naturally form positive pairs. We can categorize all positive pairs into six types according to the source of embeddings (s-spatial association, g-gene expression, and i-histology morphological) and the type of contrastive (intra- and inter-modality), further construct loss functions tailored to each category (${L}_{\mathrm{cl}}^{\mathrm{s}}$, ${L}_{\mathrm{cl}}^{\mathrm{g}}$, ${L}_{\mathrm{cl}}^{\mathrm{i}}$, ${L}_{\mathrm{cl}}^{\mathrm{sg}}$, ${L}_{\mathrm{cl}}^{\mathrm{si}}$ and ${L}_{\mathrm{cl}}^{\mathrm{gi}}$). For each mini-batch, the contrastive learning loss can be expressed by:


(8) \begin{equation*} {\displaystyle{L}_{\mathrm{cl}}=\frac{1}{2N}\sum_{k=1}^N\left[{l}_{2k-1,2k}+{l}_{2k,2k-1}\right]} \end{equation*}

Finally, the total loss of our model can be summed as follow:


(9) \begin{equation*} {\displaystyle \begin{array}{c}{L}_{\mathrm{total}}={L}_{\mathrm{recon}}+{\lambda}_1{L}_{\mathrm{cl}}^{\mathrm{s}}+{\lambda}_2{L}_{\mathrm{cl}}^{\mathrm{g}}+{\lambda}_3{L}_{\mathrm{cl}}^{\mathrm{i}}+{\lambda}_4{L}_{\mathrm{cl}}^{\mathrm{s}\mathrm{g}}+{\lambda}_5{L}_{\mathrm{cl}}^{\mathrm{s}\mathrm{i}}+{\lambda}_6{L}_{\mathrm{cl}}^{\mathrm{g}\mathrm{i}}\end{array}} \end{equation*}

where ${\lambda}_1$, ${\lambda}_2$, ${\lambda}_3$, ${\lambda}_4$, ${\lambda}_5$, and ${\lambda}_6$ are hyper-parameters used for controlling the contribution of the six types of contrastive learning losses. In our analysis, the parameters ${\mathrm{\lambda}}_1\sim{\mathrm{\lambda}}_6$ are all set to 0.1 according to the local grid searching on the mouse brain dataset (Supplementary Fig. S1).

### Feature fusion and spatial domain identification

TriCLFF framework can provide paired embeddings for each input modality. At the feature fusion stage, we first average the embeddings within each modality and then perform a weighted summation of the embeddings from different modalities. The final representation of each spot can be defined as follows:


(10) \begin{equation*} {\displaystyle{}Z={\bar{Z}}_s+\alpha{\bar{Z}}_g+\beta{\bar{Z}}_i} \end{equation*}

where ${\bar{Z}}_s$, ${\bar{Z}}_g$ and ${\bar{Z}}_i$ are averaged embeddings derived from the paired augmented inputs for spatial associations, gene expression levels and morphological features, $\alpha$ and $\beta$ are hyper-parameters used for controlling the contribution of the features, which are set to $\alpha =0.5,\beta =0.1$ in our analysis according to grid searching on the mouse brain dataset (Supplementary Fig. S2). We use the final embeddings $Z$ to perform spatial clustering since embeddings can provide more compact feature representation and more abundant multi-modal information. Spatial domains are identified based on the final embeddings $Z$ using mclust.

### Benchmarking of spatial domains

We compared TriCLFF with several other algorithms, including BayesSpace, SpaceFlow, DeepST, SEDR, SpaGCN, stLearn, SCANPY, GraphST, and STAGATE. For all competing methods, we used the default hyper-parameters recommended in their original papers. For the SCANPY method, we used the resolution parameter to determine the number of clusters.

The spatial domains are evaluated by two common clustering metrics. We utilized Adjusted Rand Index (ARI) for datasets with manually annotated labels and Averaged Silhouette Coefficient (SC) for datasets with only rough labels. ARI is a metric that can be used to measure the similarity between ground truth labels and results of clustering algorithms. The calculation formula for ARI is as follows:


(11) \begin{equation*} {\displaystyle \begin{array}{c}\mathrm{ARI}=\frac{\sum_{ij}\left(\begin{array}{c}{n}_{ij}\\{}2\end{array}\right)-\left[{\sum}_i\left(\begin{array}{c}{a}_i\\{}2\end{array}\right){\sum}_j\left(\begin{array}{c}{b}_j\\{}2\end{array}\right)\right]/\left(\begin{array}{c}n\\{}2\end{array}\right)}{\frac{1}{2}\left[{\sum}_i\left(\begin{array}{c}{a}_i\\{}2\end{array}\right)+{\sum}_j\left(\begin{array}{c}{b}_j\\{}2\end{array}\right)\right]-\left[{\sum}_i\left(\begin{array}{c}{a}_i\\{}2\end{array}\right){\sum}_j\left(\begin{array}{c}{b}_j\\{}2\end{array}\right)\right]/\left(\begin{array}{c}n\\{}2\end{array}\right)}\end{array}} \end{equation*}

In Eq (11), ${a}_i$ and ${b}_j$ represent the number of samples in the ${i}^{th}$ predicted cluster and the ${j}^{th}$ true cluster, respectively. ${n}_{ij}$ denotes the number of overlapping samples between the ${i}^{th}$ predicted cluster and the ${j}^{th}$ true cluster.

The SC value ranges from −1 to 1, with a higher value indicating that the sample is better matched to its own cluster and less matched to neighboring clusters. The averaged SC for all samples can be used to evalulate clustering performance. The averaged SC is represented as follows:


(12) \begin{equation*} {\displaystyle S=\frac{1}{N}\sum\limits_{i=1}^N\frac{\left({b}_i-{a}_i\right)}{\max \left({a}_i,{b}_i\right)}} \end{equation*}

In the above equation, $N$ denotes the total number of spots in a slice, ${a}_i$ denotes the average degree of dissimilarity from sample ${x}_i$ to other spots within the same cluster, and ${b}_i$ denotes the minimum average degree of dissimilarity from sample ${x}_i$ to other clusters. In our analysis, Euclidean distance is used for the dissimilarity metric between spots.

In addition to ARI and SC, we also use other metrics for the evaluation of clustering accuracy and quality. We incorporate the Normalized Mutual Information (NMI) and Adjusted Mutual Information (AMI) metrics to evaluate the spatial clustering accuracy. Meanwhile, Homogeneity (HOM) [40] and average intraclass correlation coefficient (ICC) [41] on top 50 principal components are used to evaluate the clustering quality.

In particular, we used CHAOS on Euclidean distance to assess spatial continuity where lower values indicate better. Meanwhile, Percentage of Abnormal Spots (PAS) [42] (i.e. the proportion of spots with a cluster label that is different from at least six of its 10 neighbors) is used to evaluate the HOM of spatial domains. For clarity, we provide the definition of the supplemented metrics (i.e. CHAOS, PAS, NMI, AMI, HOM, and ICC) in Supplementary Table S5. We use one-tailed student’s t-test to test if TriCLFF is significantly superior to each benchmarking method. The *P*-values are multiple corrected by Benjamini–Hochberg (B&H) method and those with adjusted *P*-values below 0.05 are considered statistically significant.

In order to verify the claims regarding robustness, we applied zero-mean Gaussian noise of different standard deviation ($\sigma$) to the nonzero elements of the expression matrix, ensuring all perturbed values remain non-negative. We then compared the clustering performance (ARI) of TriCLFF and STAGATE under the noisy conditions with one-tailed variance F-test. Given the limited number of repetitions, p-values below 0.1 are considered statistically significant in this test.

### Correlation assessment

We assessed the correlation of spatial clusters on both spatial interaction and gene expression levels. The neighborhood enrichment [43, 44] test assesses spatial interactions between clusters of spots or cells. We defined the spatial neighborhood using Delaunay triangulation over spot coordinates. Based on the resulting spatial graph, the neighborhood enrichment score between each pair of spatial clusters was calculated by comparing the observed co-occurrence of clusters within spatial neighborhoods to random expectations obtained via permutation. The final scores are reported as Z-scores, where higher values indicate stronger-than-expected spatial association and negative values indicate spatial exclusion between clusters. The formula for the neighborhood enrichment score is:


(13) \begin{equation*} {\displaystyle{E}_{ij}=\frac{O_{ij}-{\bar{R}}_{ij}}{\mathrm{\sigma} \left({R}_{ij}\right)}} \end{equation*}

where, ${E}_{ij}$ is a enrichment score for clusters $i$ and $j$, ${O}_{ij}$ is the number of observed co-occurrence in the spatial neighborhood of clusters $i$ and $j$, ${\bar{R}}_{ij}$ is the average co-occurrence under random permutations, ${R}_{ij}$ is co-occurrences of clusters $i$ and $j$ after random permutations, $\sigma \left({R}_{ij}\right)$ is the standard deviation of the random co-occurrences. At the gene expression level, we use Pearson’s correlation to evaluate the correlation between clusters. Average expression of spots in the cluster is treated as feature vector for each cluster.

### Downstream analyzation

We employed the Partition-based Graph Abstraction (PAGA) algorithm [45] to depict spatial trajectory and Wilcoxon’s log rank test to identify differentially expressed genes between spatial domains with a 1% False Discovery Rate (FDR) threshold. For the Gene Ontology (GO) functional enrichment analysis of differential genes, we employed hypergeometric test [46] where the significant level is adjusted *P*-value <0.05. B&H method is adopted for multiple test correction.

## Results

### TriCLFF improves the stability and accuracy of spatial domain identification in mouse brain

**Figure 2:**
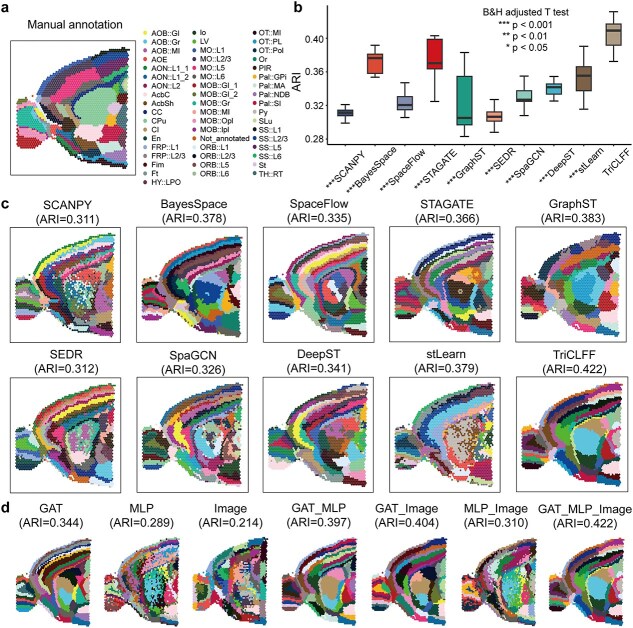
TriCLFF identifies the layers structures on mouse brain tissue anterior section. (a) Pathologic manual annotation of the mouse brain tissue anterior section. The annotation is obtained from Long Y *et al.* [34]. (b) Boxplot of clustering accuracy for the mouse brain tissue anterior section in terms of ARI values for 10 methods with each executing 20 times (one-tailed t-test adjusted by B&H method, *:$P<0.05$, **:$P<0.01$, ***:$P<0.001$, no stars: not significant). (c) Histology image and spatial domains identified by 10 different methods (SCANPY, BayesSpace, SpaceFlow, STAGATE, GraphST, SEDR, SpaGCN, DeepST, stLearn, and TriCLFF). (d) Ablation studies to investigate the contribution of different feature encoders in TriCLFF framework.

We applied TriCLFF to a 10x Visium slide from mouse brain anterior section as shown in Fig. 2. Considering the complexity of the tissue structure (including 52 categories as shown in Fig. 2a), this slide can effectively evaluate the accuracy, stability and scalability of spatial domain identification. We compared the clustering accuracy of TriCLFF with nine other identification methods (BayesSpaces, SpaceFlow, DeepST SEDR, SpaGCN, stLearn, GraphST, SCANPY, and STAGATE) with the number of clusters set to 52. We execute each algorithm 20 times to avoid the influence of random parameters (Fig. 2b) and exhibit the best results in Fig. 2c. Our proposed TriCLFF method reached the highest ARI (0.422) in the complex mouse brain anterior section, which achieved a 3.1% and 3.9% improvement than the suboptimal methods (i.e. stLearn and GraphST). We further added additional metrics including NMI, AMI, HOM, and ICC to improve confidence (Supplementary Figs S3–S6). Though the magnitude may vary, we observed that the trend of different metrics is highly consistent. In addition, TriCLFF exhibits a relatively low degree of performance degradation and good stability in the presence of noise pollution, reflecting the robustness of the algorithm (Supplementary Figs S7–S8). We have provided a benchmark on the memory and time of running for assessing scalability. The time usage is higher than other methods and the memory usage is only less than STAGATE, but both of their scales are acceptable. (Supplementary Figs S9–S10).

To further demonstrate the contribution of the three types of features, we conducted ablation studies to investigate the performance when removing some specific modalities (Fig. 2d). We found that ARI values of the frameworks with only single modality features are pretty low. Nevertheless, the GAT-based single-modal framework achieved a higher ARI score than a series of other methods such as SpaceFlow and GraphST. We also observed that the introduction of inter-modal complementary features, regardless of gene expression feature or morphological feature, can significantly improve the performance of spatial association-based spatial clustering (i.e. only use GAT encoder). When the three kinds of features are all included, the framework reached its peak performance, whose ARI has a 7.8% increment than only using spatial association feature. We also performed ablation experiments on other datasets (Supplementary Figs S11–S13) and the results are highly consistent. This evidence reveals that spatial association, gene expression level and histological morphology are all necessary information for spatial domain identification and the mechanism of contrastive learning in TriCLFF can effectively improve stability and accuracy.

### TriCLFF delivers reliable spatial domains and uncovers novel gene expression patterns in human dorsolateral prefrontal cortex

**Figure 3:**
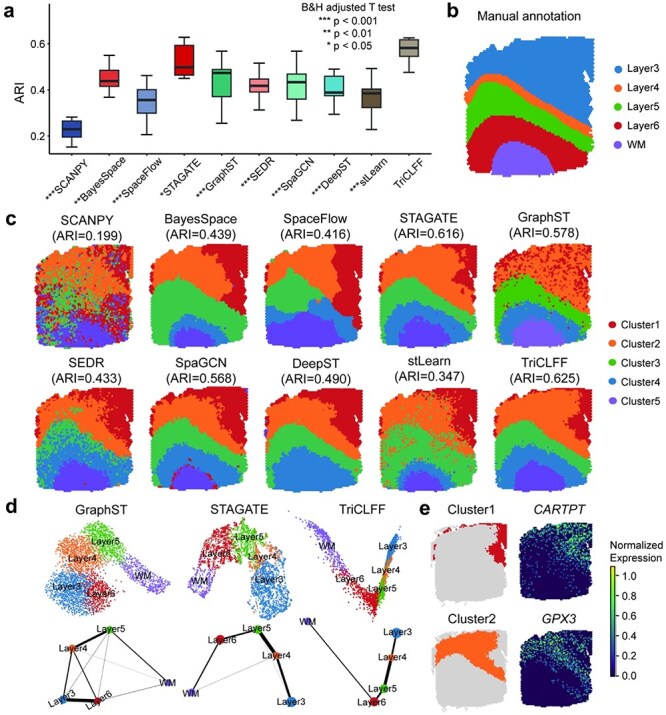
TriCLFF improves the identification of layer structures in the human DLPFC tissue. (a) Boxplot of ARI values across 12 slices of the DLPFC dataset for 10 methods. (b) Manual annotation of cortical layers and white matter (WM) in section 151 672 of DLPFC, provided by Maynard *et al.* [2]. (c) Clustering results by SCANPY, BayesSpace, SpaceFlow, STAGATE, GraphST, SEDR, SpaGCN, DeepST, stLearn, and TriCLFF on slice 151 672 (one-tailed t-test adjusted by B&H method, *:$P<0.05$, **:$P<0.01$, ***:$P<0.001$, no stars: not significant). (d) UMAP visualization and PAGA trajectory for section 151 672, using low-dimensional embeddings from three methods (GraphST, STAGATE, and TriCLFF), colors represent manual annotated layers. (e) Visualization of Cluster1 and Cluster2 from spatial domains identified by TriCLFF and the corresponding potential marker gene expressions.

To understand the reliability of TriCLFF framework, we applied it to a 10x Visium dataset with multiple slices from the human DLPFC [2] as shown in Fig. 3. Taking manual annotation as the ground truth, we compared the accuracy of TriCLFF with nine other spatial domain identification methods across the 12 slices of DLPFC dataset in Fig. 3a and Supplementary Table 2. Our proposed TriCLFF method reached the best average clustering accuracy (ARI = 0.565 $\pm$ 0.004), which achieved an 8.05% improvement over the second-best method (STAGATE). It is evident that the TriCLFF method exhibits lower variability across slices and higher cross-slice average ARI values compared to most other methods. When looking into the details, our method correctly identified white matter (WM), L5, and L6 layers in slice 151672 with a clearer boundary (Fig. 3b and c), which further reveals the value of rich cross-modality knowledge and contrastive learning mechanism in TriCLFF framework. Experiments on other slices (Supplementary Figs S14–S16) result in similar results that reveal our method has good reliability.

Though TriCLFF method improved the performance of spatial domain identification, we also observed a confusion between layer L3 and layer L4 like other methods. To explain the phenomenon, we performed UMAP visualization and PAGA trajectory analysis on embeddings learned by TriCLFF and other two suboptimal methods (GraphST and STAGATE) in Fig. 3d and Supplementary Fig. S17. The trajectory of TriCLFF showed a near-linear developmental trajectory from Layer 3 to WM, while other suboptimal methods generated trajectories with branches. In addition, UMAP visualization reveals that embeddings generated by TriCLFF method can distinguish spots in L3 and L4 generally, despite a little confusion existing. This indicates the existence of inconsistent gene expression patterns with histological annotations. Hence, we observed the spatial distribution of multiple genes and found expression patterns of *CARTPT* and *GPX3* are highly consistent with the Cluster1 and Cluster2 detected by TriCLFF (Fig. 3e), which has become powerful evidence supporting the assumption. These phenomenon reveals TriCLFF can help to identify spatial domains with more biological significance.

### TriCLFF enables cross-platform spatial domain identification and discovers novel layer-specific marker genes in mouse olfactory bulbs

**Figure 4:**
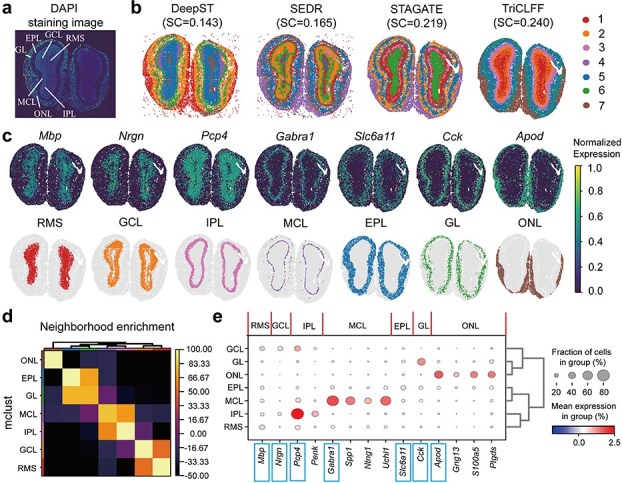
TriCLFF demarcates the laminar structure in mouse olfactory bulb. (a) Rough annotation of laminar organization of the mouse olfactory bulb on DAPI-stained image, provided by Chen A [14]. (b) Spatial domains identified by DeepST, SEDR, STAGATE, and TriCLFF. (c) Visualization of the spatial domains identified by TriCLFF. Normalized expression of previously reported layer marker genes (*Mbp*, *Nrgn*, *Pcp4*, *Gabra1*, *Slc6a11*, *Cck*, and *Apod*) are displayed as reference. (d) Heatmap of neighborhood enrichment scores between identified spatial domains. Each value represents the enrichment z-score of spatial proximity between a pair of clusters, higher scores indicate stronger spatial interaction. (e) Highly expressed DEGs in each layer. Previously reported marker genes were marked by boxes.

Different spatial transcriptomic platforms possess distinct technical advantages. Hence, we evaluated the spatial domains identified by TriCLFF on a mouse olfactory bulb tissue Stereo-seq slide [14] (Fig. 4). As depicted in the 4′,6-Diamidino-2-Phenylindole (DAPI) staining image [17] (Fig. 4a), the tissue exhibits a laminar structure comprising various layers including the olfactory nerve layer (ONL), glomerular layer (GL), external plexiform layer (EPL), mitral cell layer (MCL), internal plexiform layer (IPL), granule cell layer (GCL), and rostral migratory stream (RMS). Since there is no spot-level fine-grained annotation, we use a widely used unsupervised evaluation metric, SC, to evaluate the spatial domain identification performance. Our proposed TriCLFF method achieved the highest SC (0.240) among all the methods tested (Fig. 4b). Considering SC is not sufficient to evaluate spatial association between domains, we further compared spatial domains and expression distribution of previously known layer marker genes (RMS-Mbp, GCL-Nrgn, IPL-Pcp4, MCL-Gabra1, EPL-Slc6a11, GL-Cck, ONL-Apod) in Fig. 4c and investigated neighborhood enrichment between domains in Fig. 4d by permutation test. The one-to-one correspondence between TriCLFF identified domains and known layer-specific markers (Fig. 4c) confirms that the embeddings capture structural-functional information consistent with previous biological knowledge. The enrichment scores and heatmap in Fig. 4d offer interpretable insights into the spatial organization of tissue layers. High enrichment scores between RMS and GCL, IPL and MCL, as well as EPL and GL prove that the clustering results of our algorithm are more consistent with the previous studies and can accurately demarcate the laminar structure. We found that TriCLFF-detected clusters are weakly overlapped and highly consistent with the expression distribution of the marker genes spatially, even in the narrow regions such as MCL. These results reveal that TriCLFF method is an available solution for layer annotation on Stereo-seq platform and confirms its exceptional cross-platform capability.

We further discovered the potential of layer marker gene detection based on the accurate layer annotation provided by TriCLFF. Highly expressed differential expression genes (DEGs) on each layer are filtered by Wilcoxon signed-rank test (B&H FDR<0.01, logFC>1) and most of the known layer marker genes (6 of 7) are significantly expressed in their corresponding layers (Genes framed in blue in Fig. 4e). In addition, our analysis revealed that *Ptgds*, *S100a5*, and *Gng13* are highly expressed in the ONL layer, while *Ntng1*, *Spp1*, and *Uchl1* were found to be highly expressed in the MCL layer and *Penk* is identified as a highly expressed gene in the IPL layer (Supplementary Figs S18–S20). From the view of gene function, *Ptgds*, *S100a5*, and *Gng13* plays a crucial role in maintaining function, signal transduction, and protecting and regenerating olfactory nerve fibers [47–49]; *Ntng1*, *Spp1*, and *Uchl1* regulate neuronal excitability, synaptic plasticity, energy metabolism, and intercellular connectivity to ensure precise processing and efficient transmission of olfactory signals [50–52]; *Penk* encodes enkephalin precursors that modulate inhibitory interneuron activity and synaptic transmission. Its expression in IPL likely reflects its role in fine-tuning olfactory signal flow through regulating GABAergic inhibition between mitral and granule cells, supporting the functional specificity of the spatial domains identified by TriCLFF [53]. Functions of the genes are highly supporting the important role of spindle cells in olfactory signal processing, which indicates these unreported genes might be powerful potential biomarkers.

### TriCLFF reveals tissue functional heterogenicity in human breast cancer with finer-grained spatial domain identification

**Figure 5:**
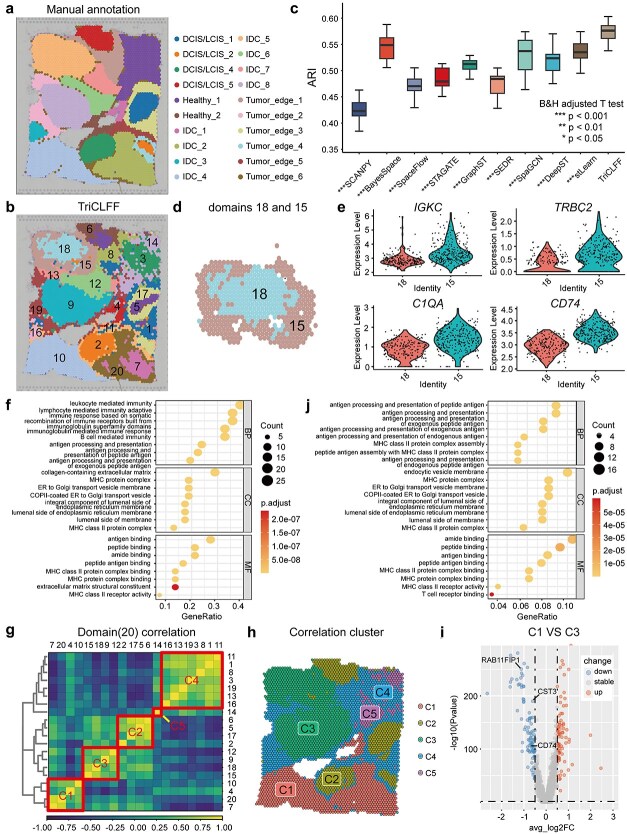
TriCLFF reveals intratumoral spatial heterogeneity in human breast cancer. (a) Manual annotation of the breast cancer sample, provided by Long Y *et al.* [34]. (b) Spatial domains identified by TriCLFF, where each domain is labeled with the corresponding region label. (c) Boxplot of clustering accuracy of the human breast cancer dataset in terms of ARI values for ten methods with each executing 20 times (one-tailed t-test adjusted by B&H method, *:$P<0.05$, **:$P<0.01$, ***:$P<0.001$, no stars: not significant). (d) TriCLFF divides the tumor region IDC_5 into an outer ring (domain 15) and a tumor core (domain 18). (e) *IGKC*, *TRBC2*, *C1QA*, and *CD74* express differentially between domains 18 and 15. (f) The GOEA of the DEGs between domains 18 and 15. (g) Heatmap of Pearson correlation coefficient between domains (domains = 20). (h) Five regions were divided according to the domain correlation results and hierarchical clustering method at a basis of accurately dividing IDC and DCIS/LCIS regions. (i) Volcano graph of DEGs between regions C1 and C3. (j) GOEA of DEGs between C1 and C3 regions.

Functional discrepancy is an important facet to depict the quality of spatial domains. We used a human breast cancer slide on 10x Visium platform to reveal the functional heterogeneity of pathological tissue (Fig. 5). The tissue can be roughly categorized into four main morphotypes [i.e. ductal carcinoma *in situ*/lobular carcinoma *in situ* (DCIS/LCIS), healthy tissue, invasive ductal carcinoma (IDC), and tumor edge] and previously be divided into 20 regions by Long *et al.* [34] as shown in Fig. 5a. Figure 5b shows the spatial domain identification results of the TriCLFF method. Through 10 repeating tests, TriCLFF achieved the highest ARI median (0.5665, Fig. 5c and Supplementary Fig. S21). In addition, spatial domains identified by TriCLFF has approximate or better regional continuity (Supplementary Figs S22–S23), which validated the stability and accuracy again.

When looking into the clusters, we found TriCLFF divided tumor region IDC_5 into an outer ring (domain 15) and a tumor core (domain 18) while most other comparison methods divided the region into relatively disordered three parts (Fig. 5d and Supplementary Fig. S21). Herein, we detected DEGs between domains 18 and 15 and analyzed their function by Gene Ontology enrichment analysis (GOEA). Through a relatively relaxed criterion (|logFC|≥0.5 and B&H FDR<0.05), we found 72 DEGs in total (Supplementary Fig. S24), including *IGKC*, *TRBC2*, *C1QA* and *CD74*, which are highly expressed in domain 15 (Fig. 5e). As crucial indicators of tumor metastasis and immune responses in breast cancer [54–58], the high expression of these genes in region 15 signifies active immune responses aimed at recognizing and eliminating tumor cells. Conversely, the DEG expression patterns observed in region 18 suggest a greater potential for immune evasion within this area, creating an immunosuppressive microenvironment that hampers effective immune clearance. Additionally, the enriched GO terms of these DEGs predominantly focused on the functions related to leukocyte mediated immunity, collagen-containing extracellular matrix, integral component of lumenal side of endoplasmic reticulum membrane and antigen binding (Fig. 5f). These results indicate significant differences in the microenvironment and immune responses between the central and outer ring regions of the tumor, reflecting the adaptive and invasive characteristics. Moreover, TriCLFF can also help to identify another group of finer regions, regions 5 and 17 (Supplementary Figs S25–S27), with similar spatial distribution and functional differences. These phenomena reveal that TriCLFF can identify finer domains with meaningful biological functions.

To further investigate the contribution of TriCLFF on global spatial heterogeneity revealing of tumors, we calculated Pearson correlation coefficient among the 20 domains (Fig. 5g) and further merged them into 5 regions (Fig. 5h), including two IDC regions (C1 and C3), DCIS/LCIS (C2), healthy tissue (C5), and tumor edge (C4), utilizing hierarchical clustering method at a basis of accurately dividing IDC and DCIS/LCIS regions. To quantify the consistency between our merged spatial regions and histological annotations, we further integrated C1 and C3 due to their substantial overlap with the IDC region, while retaining the other three regions to ensure consistency between the number of merged regions and histological types. The ARI score between merged regions with manual annotations is 0.625 (Supplementary Figs S28–S29). Specifically, in Fig. 5g, the IDC regions (C1 and C3) are closely adjacent with DCIS/LCIS region (C2) while the Tumor edge region (C4) is closer to normal tissue (C5), reflecting the heterogeneity at transcriptomic level between tumors and tumor edges. Additionally, the path from C5 through C2, C3 and C1 illustrates the developmental trajectory of normal tissue, carcinoma *in situ* and invasive carcinoma. Since TriCLFF divided the IDC domains into two regions (C1 and C3), we further discovered the functional differences between them. We first performed differential expression analysis and detected 171 DEGs (|logFC|≥0.5 and B&H FDR<0.05) while 79 of them are up-regulated in region C1 (Fig. 5i). Among the DEGs, we detected some marker genes (*CD74*, *RAB11FIP1*, and *CST3*) related to tumor invasion [57, 59, 60] which are highly expressed in region C3 (Supplementary Fig. S30). This suggests a difference in the degree of invasion and malignancy between the two regions. GOEA indicates that detected DEGs are enriched in pathways related to antigen processing and presentation of peptide antigens, endocytic vesicle membranes, and amide binding (Fig. 5j), which reveals the difference between the two regions at immune and metabolic levels. Interestingly, when observing the IDC regions (C1 and C3) and DCIS/LCIS region (C2), we found most of the DEGs are down regulated in IDC regions (Supplementary Figs S31–S32). The DEGs between C3 and C2 are mainly enriched on immune-related functions such as Major Histocompatibility Complex (MHC) class II antigen processing and presentation, while DEGs between C1 and C2 enriched on metabolism and development-related functions such as response to nutrient levels and skin development (Supplementary Figs S33–S34). These results support the inference that the two IDC regions have significant functional heterogeneity and the finer-grained spatial domain identification provided by TriCLFF can help to reveal this kind of discovery.

## Discussion

In ST data, accurate identification of spatial domains is crucial for understanding the organization, function, and disease progression in heterogeneous tissues. In this work, we proposed TriCLFF, a multi-modal feature fusion framework to generate low-dimensional spot embeddings for spatial domain identification. The framework employs a contrastive learning approach to seamlessly integrate spatial association, gene expression, and histological morphological features that have been previously extracted by graph attention autoencoders, MLP, and Swin Transformer, respectively. This integration facilitates the extraction of complementary information among these modalities, while reducing noise interference and information redundancy. As a result, it improves the feature representation capability of the model and reduces the risk of overfitting, further improving overall learning efficiency and accuracy.

We performed intensive experiments on multiple datasets from 10x Visium and Stereo-seq platforms to validate the value of TriCLFF framework on both technical and biological levels. At the technical level, we discovered TriCLFF can significantly improve the spatial domain identification performance and provide enhanced ability of generalization and stability in unsupervised scenes, regardless of the platforms. The technical advantages of our proposed framework are derived from two key considerations: the incorporation of comprehensive input information and the extraction of complementary fusion features. The ablation studies demonstrate that spatial association, gene expression, and histological morphology are all essential information for spatial domain identification applications, while gene expression information is the core factor to determine the spatial domains. Moreover, the integration of multiple encoders with contrastive learning can effectively compensate for limitations of feature extraction in single modal and utilize complementary among modalities. For multi-modal features, TriCLFF projects them into a shared latent space to align them and further fuse them with weighted summation method. This enables the proportion of each modality in the final embeddings to be dynamically adjusted according to the actual situation. In addition, this strategy is computational-friendly since there is no trainable structure for feature fusion. Therefore, we assert that the TriCLFF framework serves not only as a spatial domain identification method but also offers fundamental principles and guidelines for subsequent methodologies.

With the advantage at technical level, TriCLFF is capable of delivering valuable and innovative biological discoveries. Our experiments confirmed that TriCLFF is a powerful tool to reveal the developmental trajectories of tissues, detect unknown gene expression spatial patterns, discover novel biomarker genes, and identify functional domains of pathological tissues. In the analysis of DLPFC dataset, we depicted linear developmental trajectory from Layer 3 to WM and discovered novel gene expression spatial patterns represented by *CARTPT* and *GPX3*. The role of *CARTPT* gene and its encoded CART peptide in the central nervous system is mainly reflected in the regulation of neurotransmission and neurobehavior [61], while *GPX3* is vital in regulating metabolism and facilitating signal transduction [62]. Revealing such unique spatial patterns of gene expression may provide novel prospects for functional analysis. For the olfactory bulb data, we detected seven genes that are highly expressed in ONL (*Ptgds*, *S100a5*, and *Gng13*), MCL (*Ntng1*, *Spp1*, and *Uchl1*) and IPL (*Penk*) layers. The functioning of the genes highly supports the important role of spindle cells in olfactory signal processing. The primary objective may not be focused on, but we posit that these seven genes have the potential to serve as marker genes for corresponding layers. When focusing on the breast cancer data, we observed the differences in immune and metabolic levels within IDC regions, both globally and locally. The discovery can offer valuable insights for critical applications like tumor classification and pharmaceutical testing. All the discoveries above are sourced from the accurate and finer-grained spatial domain identification, which proved the value of TriCLFF framework at a biological level.

Despite the remarkable performance achieved, there is still room for improvement in the TriCLFF framework, particularly in terms of enhancing the richness of input data and refining feature extraction. For the richness of input data, even the ablation studies have demonstrated the necessity of spatial association, gene expression, and histological morphology information, the emergence of novel spatial omics technology (such as the spatial proteomics [63]) may further improve spatial domain identification performance. In addition, vertical data integration of adjacent tissue sections and cellular level spatial resolution may also provide novel prospects for the application. On the other hand, the ImageNet pretrained Swin Transformer module we use may potentially compromise the precision of histological feature extraction due to the huge gap between training and testing data. Employing a histology image pretrained or fine-tuned module may further improve the overall performance. Given the significance of interpretability and biological relevance of the embeddings, future work can propose techniques to highlight the factors that drive the model’s decisions.

## Conclusion

In response to the need for spatial domain identification in ST, focusing on the richness and complementarity of feature extraction, we propose a novel cross-modal feature fusion framework called TriCLFF in this study. The framework employs a diverse range of encoders, including GATE, MLP, and Swin Transformer, to extract comprehensive gene expression features, associations with spatial locations, and histological morphology information from spatial transcriptome profiles and pathological images. Additionally, it utilizes contrastive learning strategies to explore the complementarity of multi-modal features. The validation experiments conducted on multiple datasets demonstrate that TriCLFF can significantly enhance the accuracy of spatial domain identification and effectively reveal the associations between spatial domains and biological functions. When combined with advanced downstream analysis techniques, the TriCLFF framework is expected to provide robust technical support for critical applications such as tumor microenvironment analysis, disease classification, and drug screening.

**Key Points**

- Identifying Spatial domains with similar expression patterns and histological features is essential to depict complex spatial structures and functions of cells and tissues.
- TriCLFF is a contrastive learning-based multi-modal feature fusion framework for spatial domain identification in spatial transcriptomics.
- TriCLFF effectively integrates spatial association, gene expression and morphological features, offers an improved accuracy and stability in spatial domain identification tasks.
- TriCLFF can identify finer-grained spatial domains and unveil previously unknown gene expression patterns, providing novel insights for understanding tissue functions.

## Supplementary Material

supplementary-material_bbaf316

## Data Availability

All data analyzed in this paper are available in raw form from their original sources. Specifically, the DLPFC dataset is accessible (https://github.com/LieberInstitute/HumanPilot). The MouseBrain dataset and human breast cancer data can be obtained at https://zenodo.org/record/6925603#.YuM5WXZBwuU. The processed Stereo-seq data from mouse olfactory bulb tissue is accessible at https://github.com/JinmiaoChenLab/SEDR_analyses.

## Code availability

The source code of TriCLFF is available online at https://github.com/HBZZ168/TriCLFF.
