# Supplementary material for: TriCLFF: a multi-modal feature fusion framework using contrastive learning for spatial domain identification
Source: Brief Bioinform. 2025 Jul 10;26(4):bbaf316. doi: 10.1093/bib/bbaf316 (PMC12245166; doi:10.1093/bib/bbaf316)
Supplement: supplementary-material_bbaf316 [file supplementary-material_bbaf316.zip › Supplementary_FigureS1-S34(1)_bbaf316.docx]

**Supplementary Information**

**TriCLFF: A multi-modal feature fusion framework using contrastive learning for spatial domain identification**

**Supplementary Figure S1.** Local grid searching of parameters $\lambda_{1}\sim\lambda_{6}$. Parameters are adjusted one-by-one from 0 to 0.6 at a step length of 0.1 and fix all the other parameters to 0.1 on the mouse brain data.

**Supplementary Figure S2.** Local grid searching of parameters $\alpha$ and $\beta$. Parameters are adjusted from 0 to 1 at a step length of 0.1 on the mouse brain data.

**Supplementary Figure S3.** Boxplot of clustering accuracy in all methods in terms of Normalized Mutual Information (NMI) values. One-tailed student’s t-test result shows that our proposed TriCLFF is significantly superior to each benchmarking method.

**Supplementary Figure S4.** Boxplot of clustering accuracy in all methods in terms of Adjusted Mutual Information (AMI) values. One-tailed student’s t-test result shows that our proposed TriCLFF is significantly superior to each benchmarking method.

**Supplementary Figure S5.** Boxplot of clustering quality in all methods in terms of Homogeneity (HOM) values. One-tailed student’s t-test result shows that our proposed TriCLFF is significantly superior to each benchmarking method.

***
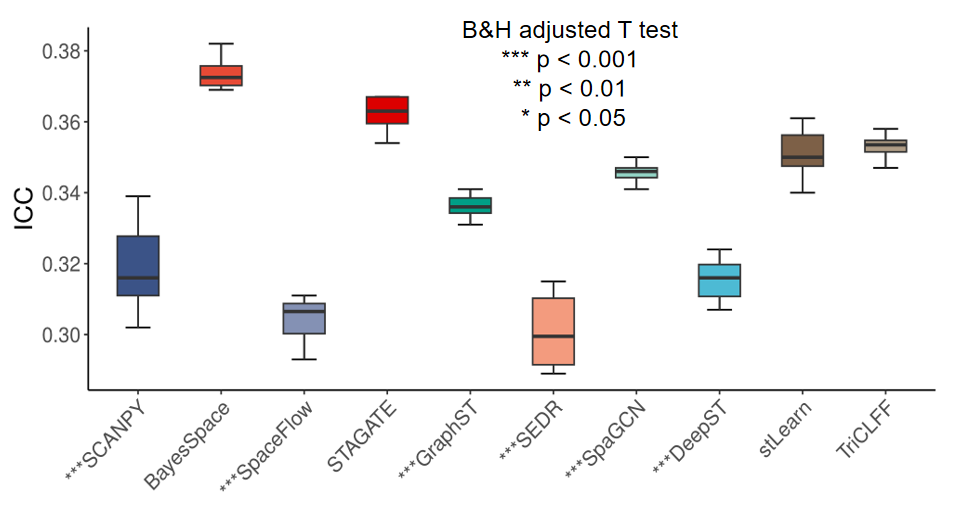
***

**Supplementary Figure S6.** Boxplot of clustering quality in all methods in terms of intraclass correlation coefficient (ICC) values.


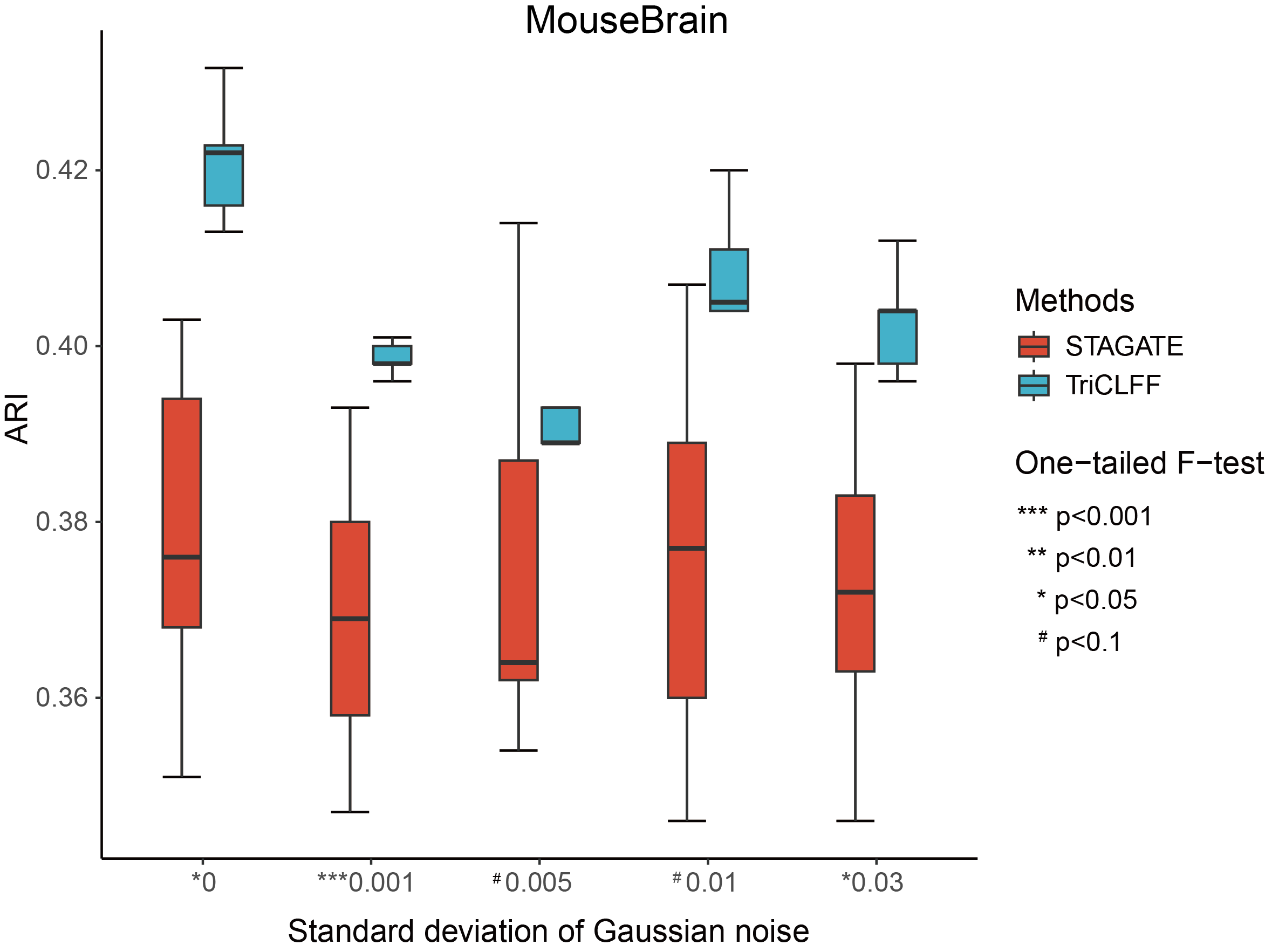


**Supplementary Figure S7.** Simulated noisy datasets with Gaussian noise of different standard deviation ($\boldsymbol{\sigma}$) were constructed on mouse brain data. One-tailed F-tests confirmed the statistical significance of the performance difference, indicating that compared with STAGATE, TriCLFF has stronger robustness and stability.

**
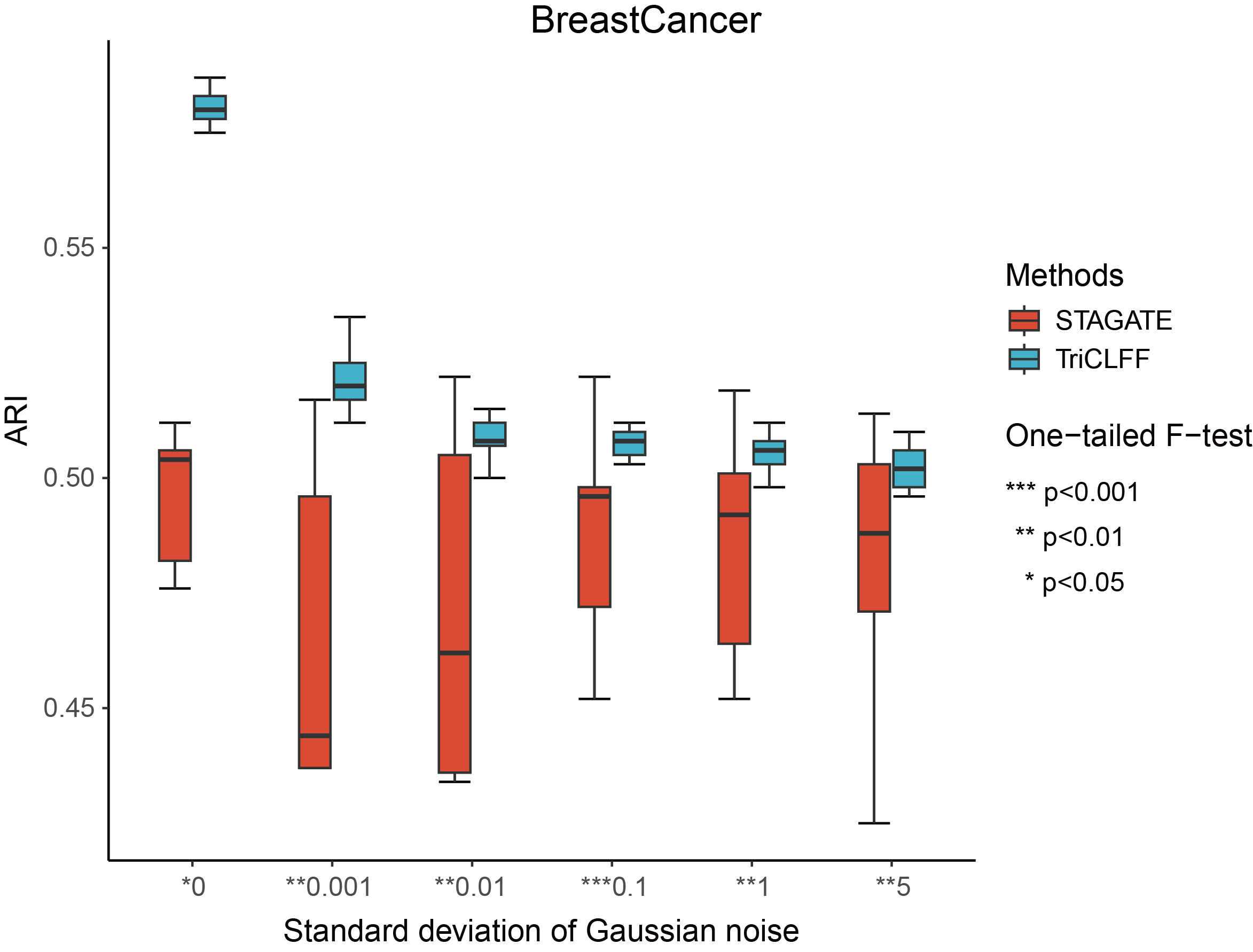
**

**Supplementary Figure S8.** Simulated noisy datasets with Gaussian noise of different standard deviation ($\boldsymbol{\sigma}$) were constructed on breast cancer data. One-tailed F-tests confirmed the statistical significance of the performance difference, indicating that compared with STAGATE, TriCLFF has stronger robustness and stability.


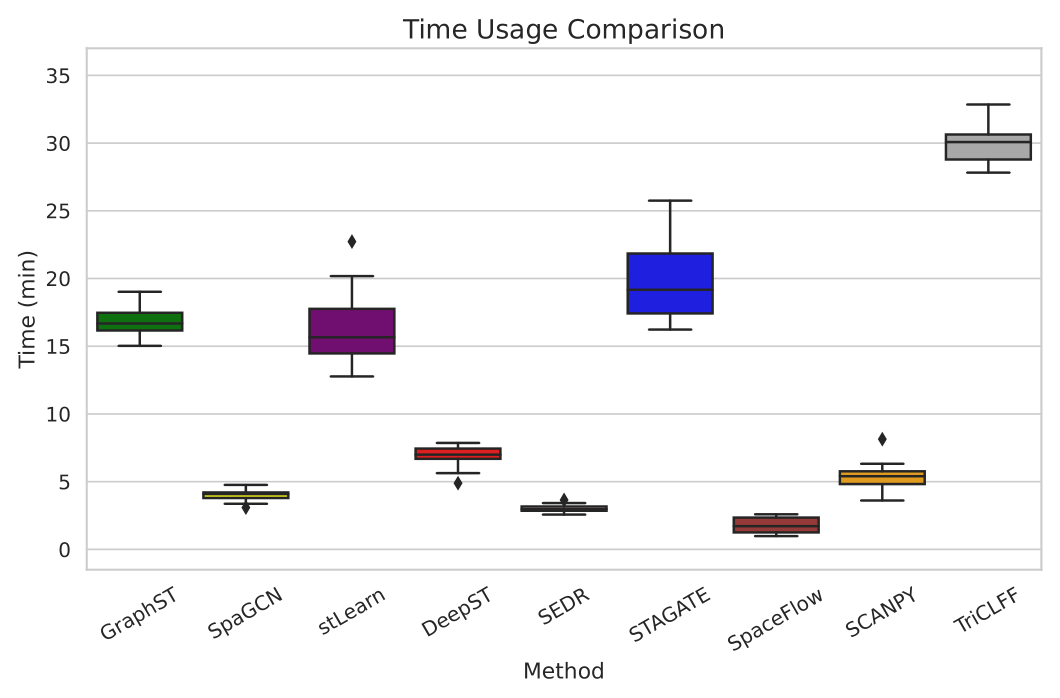


**Supplementary Figure S9.** Time cost of TriCLFF and other eight python-based methods.


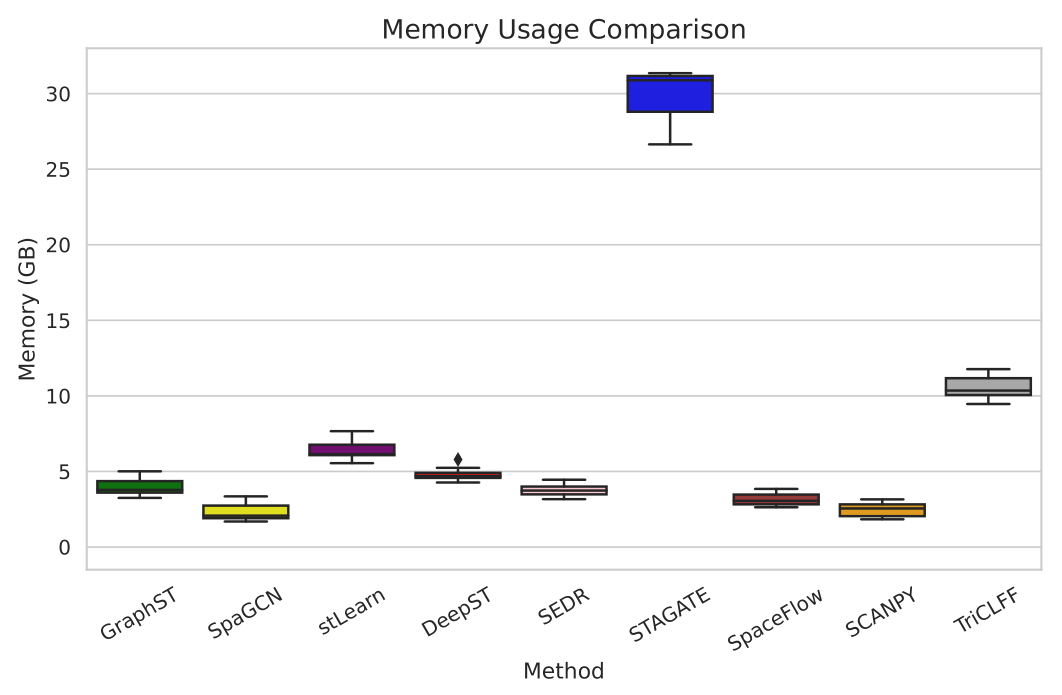


**Supplementary Figure S10.** Memory usage of TriCLFF and other eight python-based methods.


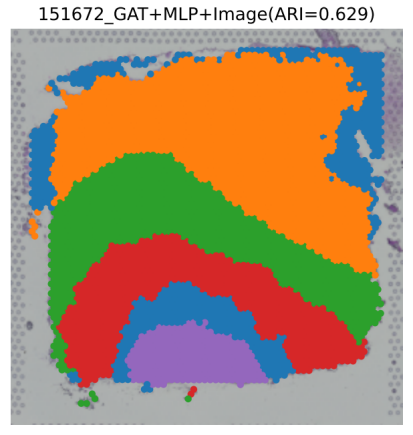

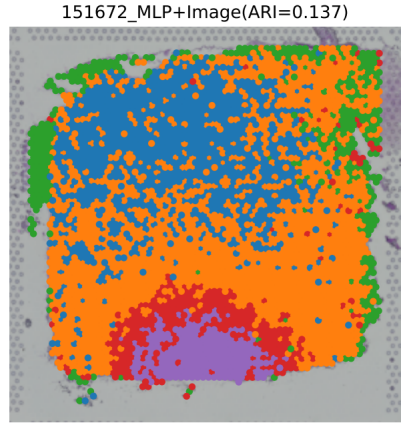

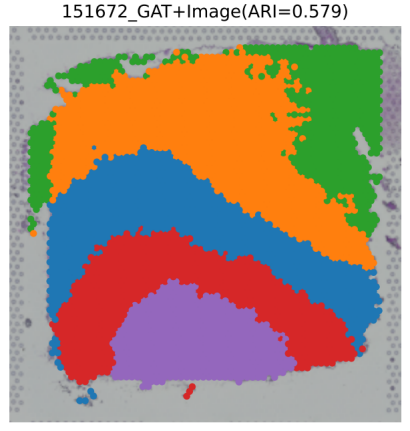

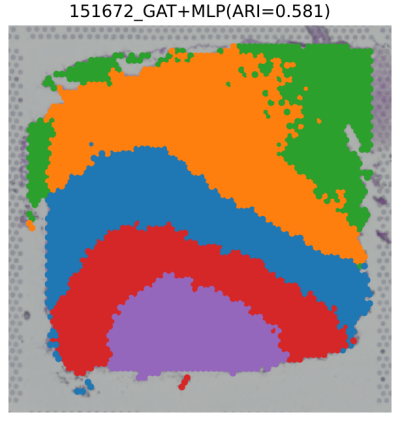

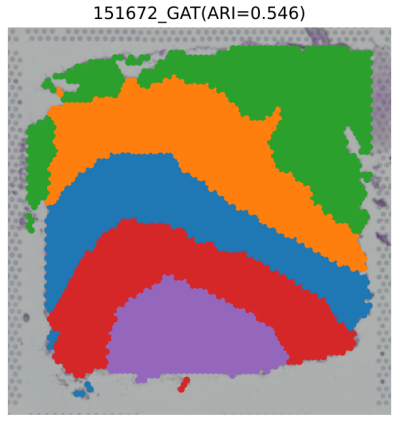


**Supplementary Figure S11.** Comparison of ablation experimental results of the TriCLFF method on section 151672 of human dorsolateral prefrontal data. In total, ablation comparisons were performed for five cases, including GAT+MLP+Image where the graph attention self-encoder, MLP, and Image were all used, and GAT+MLP represented GAT and MLP were applied and Image was not included. There was GAT+Image case where GAT and Image were applied and MLP was not included. There was the case MLP+Image, which applied MLP and Image without GAT, and the case GAT where only GAT was applied.


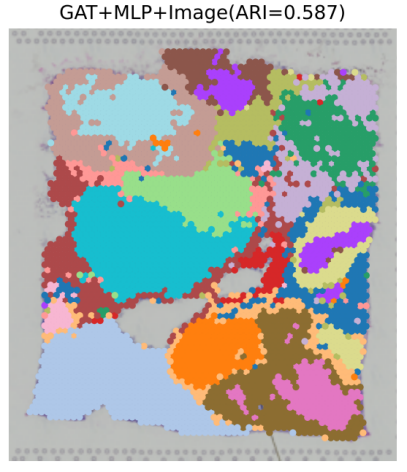

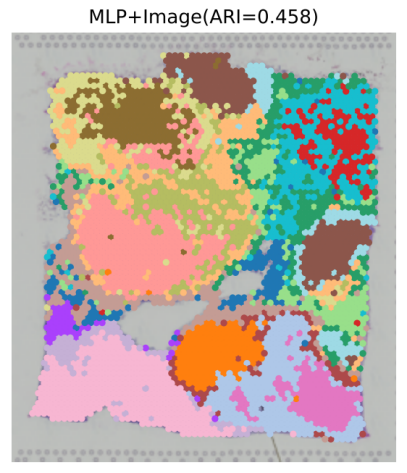

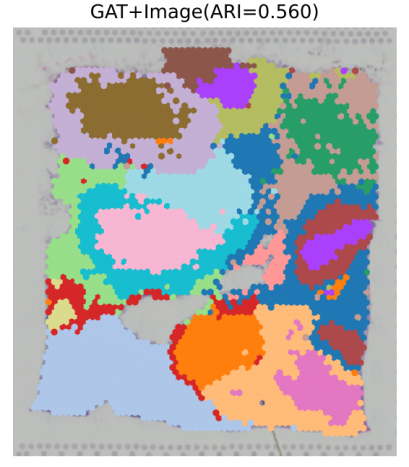


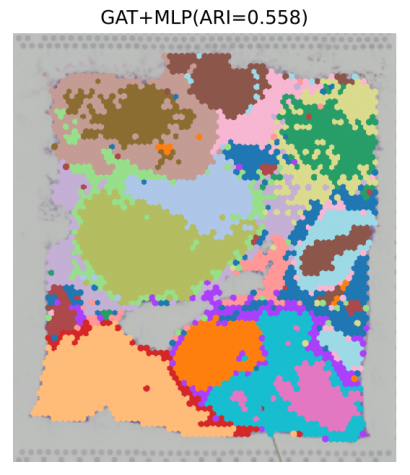

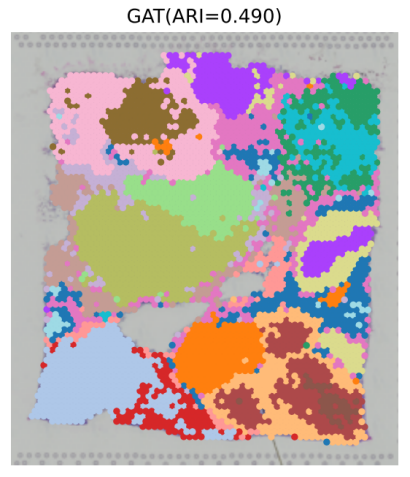


**Supplementary Figure S12.** Comparison of ablation experimental results of the TriCLFF method on the human breast cancer dataset. In total, ablation comparisons were performed for five cases, including GAT+MLP+Image where the graph attention self-encoder, MLP, and Image were all used, and GAT+MLP where GAT and MLP were applied and Image was not included. There was GAT+Image case where GAT and Image were applied and MLP was not included. There was the case MLP+Image which applied MLP and Image without GAT, the case GAT where only GAT was applied.


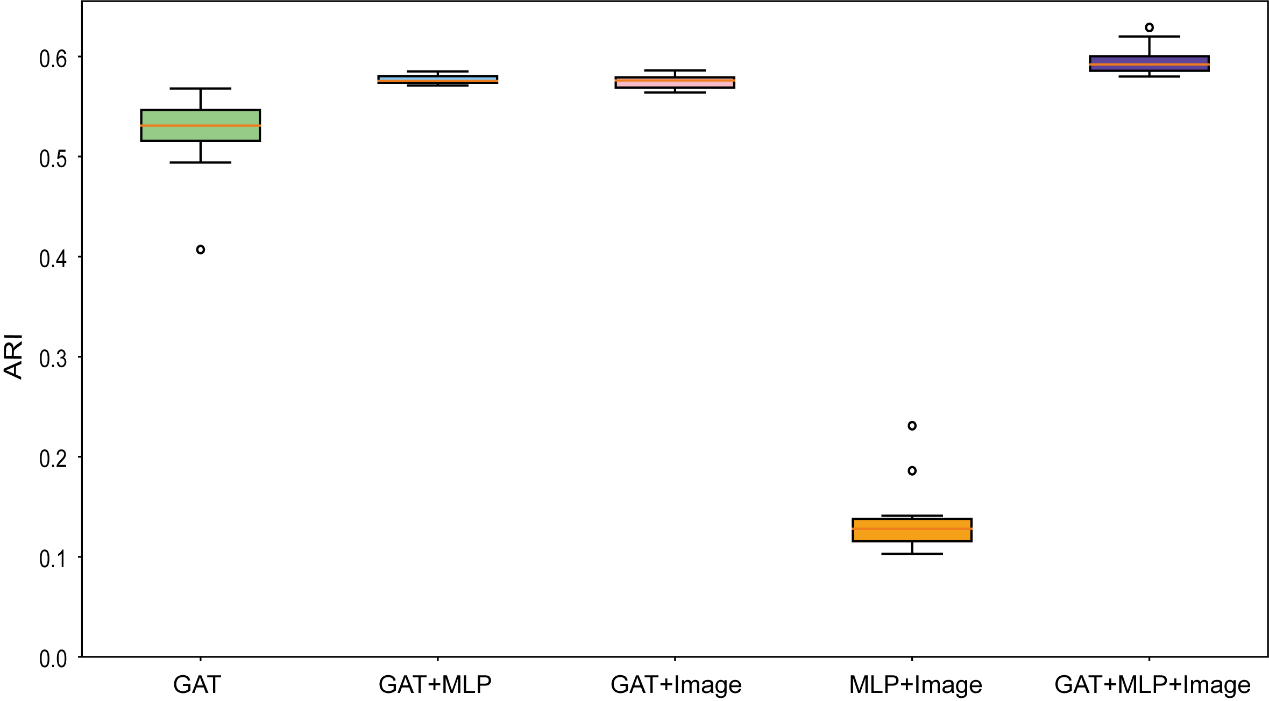


**Supplementary Figure S13.** Boxplot of ARI scores from the ablation experiments conducted on slice 151672 of the DLPFC dataset.


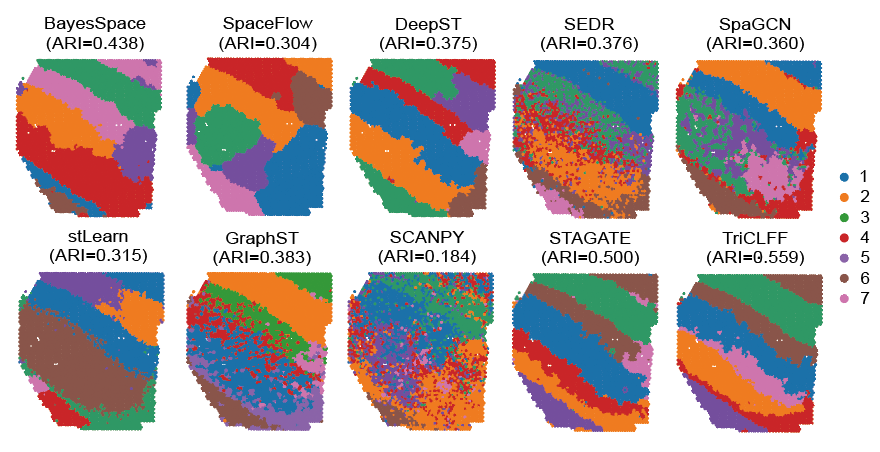


**Supplementary Figure S14.** Cluster results generated by BayesSpace, SpaceFlow, DeepST, SEDR, SpaGCN, stLearn, GraphST, SCANPY, STAGATE and TriCLFF in the DLPFC section 151508.


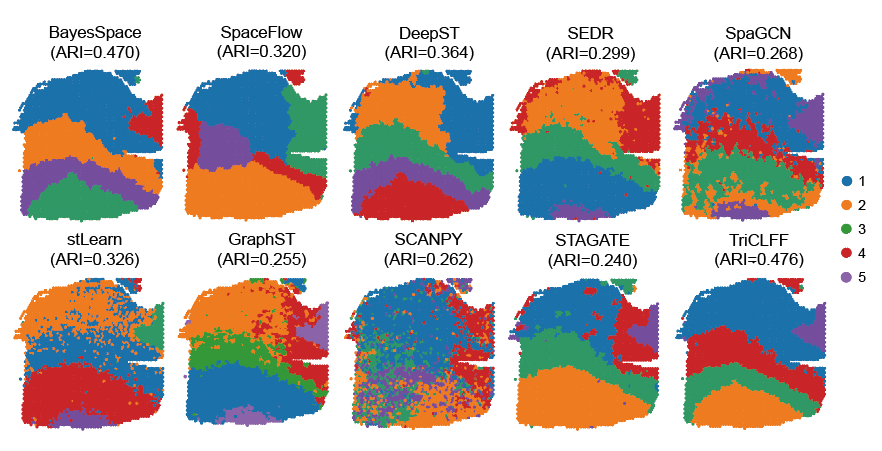


**Supplementary Figure S15.** Cluster results generated by BayesSpace, SpaceFlow, DeepST, SEDR, SpaGCN, stLearn, GraphST, SCANPY, STAGATE and TriCLFF in the DLPFC section 151669.


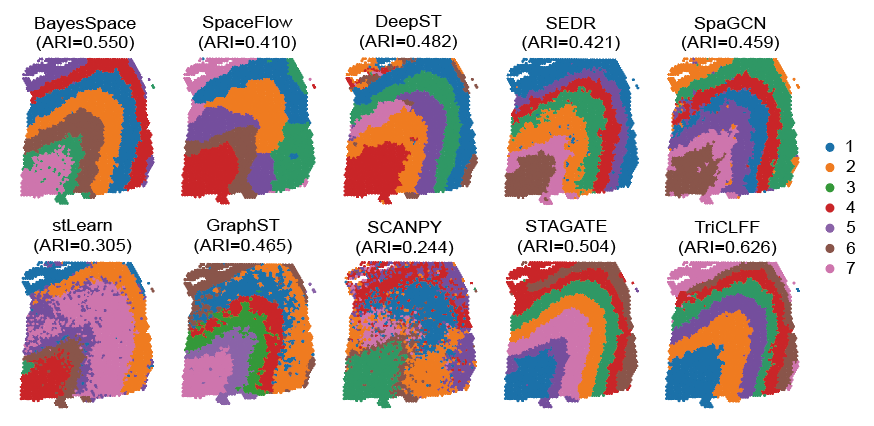


**Supplementary Figure S16.** Cluster results generated by BayesSpace, SpaceFlow, DeepST, SEDR, SpaGCN, stLearn, GraphST, SCANPY, STAGATE and TriCLFF in the DLPFC section 151673.


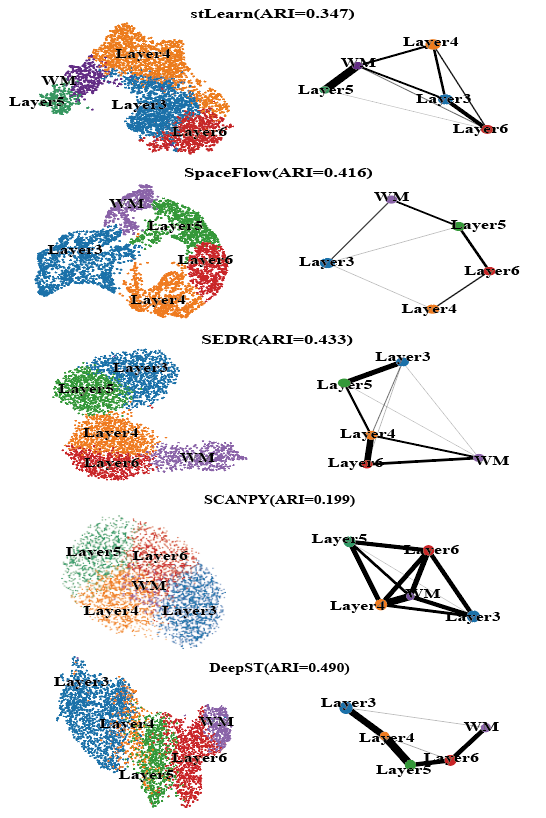


**Supplementary Figure S17.** The UMAP visualizations and PAGA trajectory of section 151672 of the human dorsolateral prefrontal cortex (DLPFC) from stLearn, SpaceFlow, SEDR, SCANPY and DeepST, respectively.


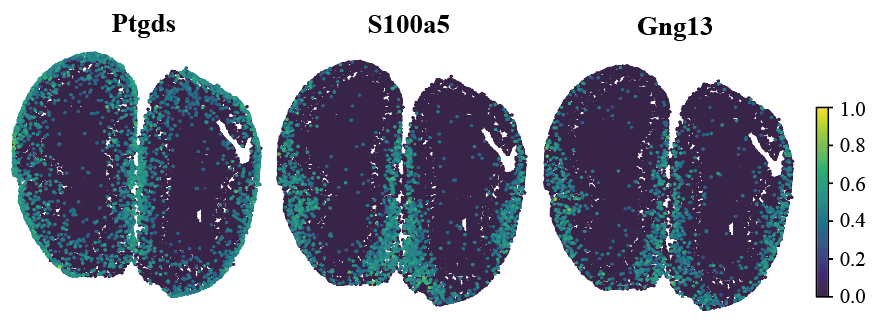


**Supplementary Figure S18.** Ptgds, S100a5, and Gng13 are specifically highly expressed in the ONL layer of mouse olfactory bulb.


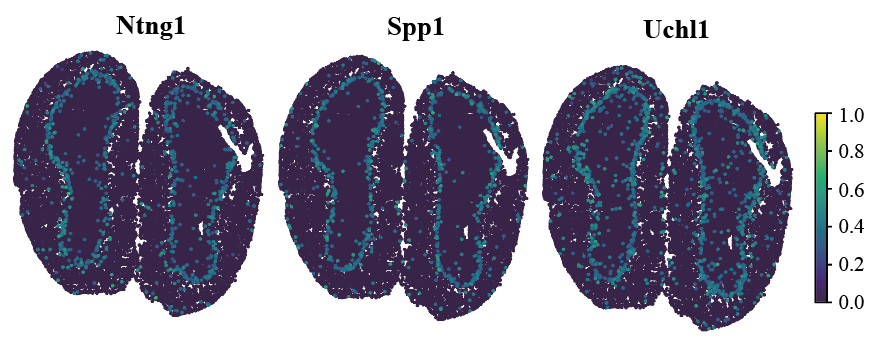


**Supplementary Figure S19.** Ntng1, Spp1, and Uchl1 are specifically highly expressed in the MCL layer of mouse olfactory bulb.

**
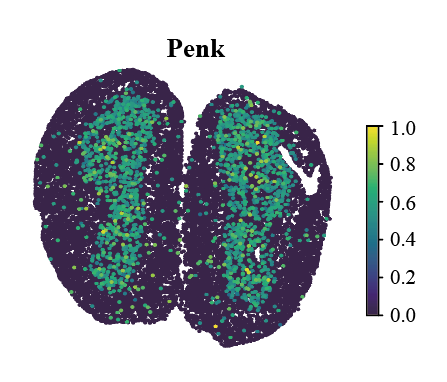
**

**Supplementary Figure S20.** Penk is specifically highly expressed in the IPL layer of mouse olfactory bulb.


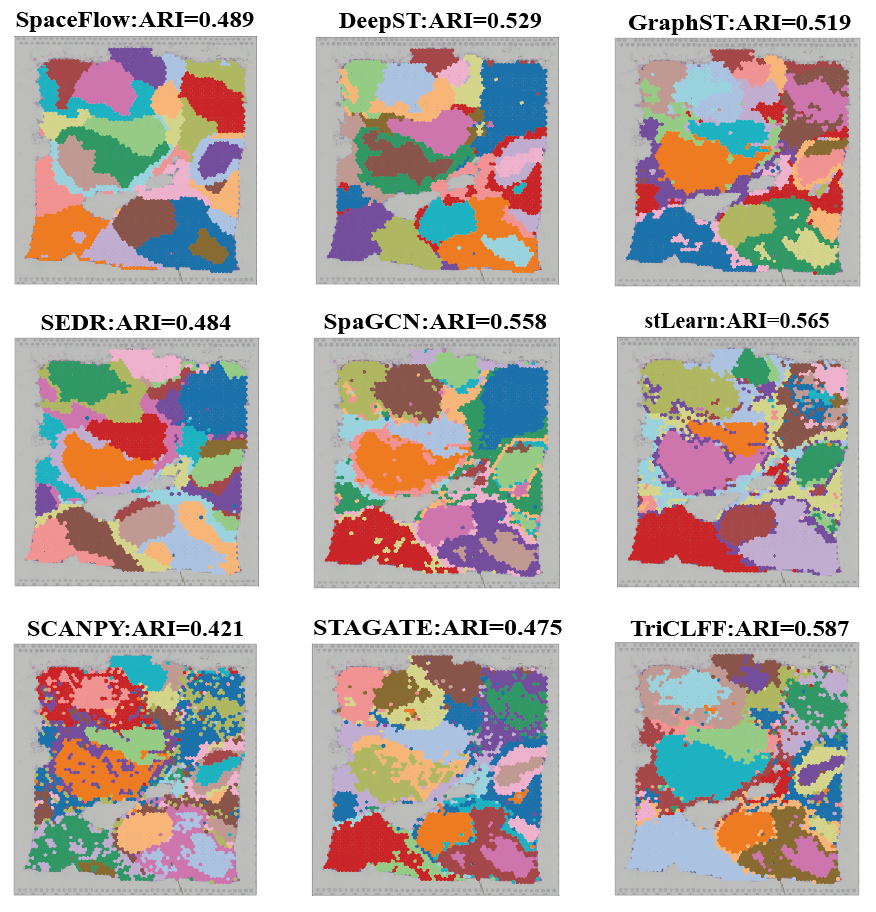


**Supplementary Figure S21.** Spatial domains identified by nine different methods (SpaceFlow, DeepST, GraphST, SEDR, SpaGCN, stLearn, SCANPY, STAGATE, and TriCLFF) on human breast cancer.

***
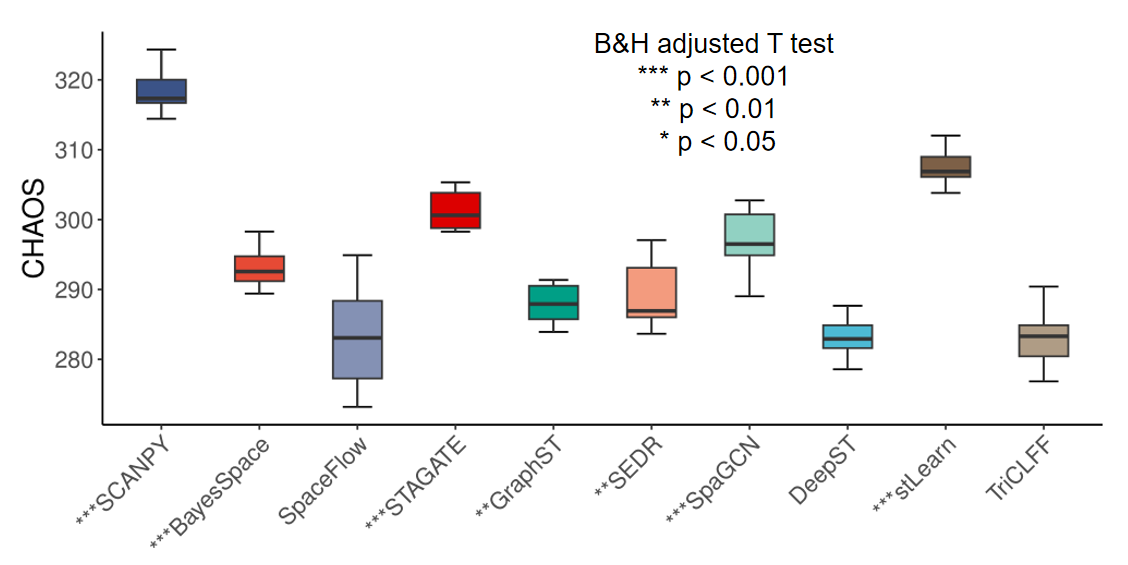
***

**Supplementary Figure S22.** Spatial continuity evaluated by CHAOS on breast cancer data. Stars near the name of method indicate statistical significance of TriCLFF superior to the corresponding method. (one-tailed t-test adjusted by Benjamini-Hochberg method, *:$\boldsymbol{p}<\mathbf{0}.\mathbf{05}$, **:$\boldsymbol{p}<\mathbf{0}.\mathbf{01}$, ***:$\boldsymbol{p}<\mathbf{0}.\mathbf{001}$, no stars: not significant)

***
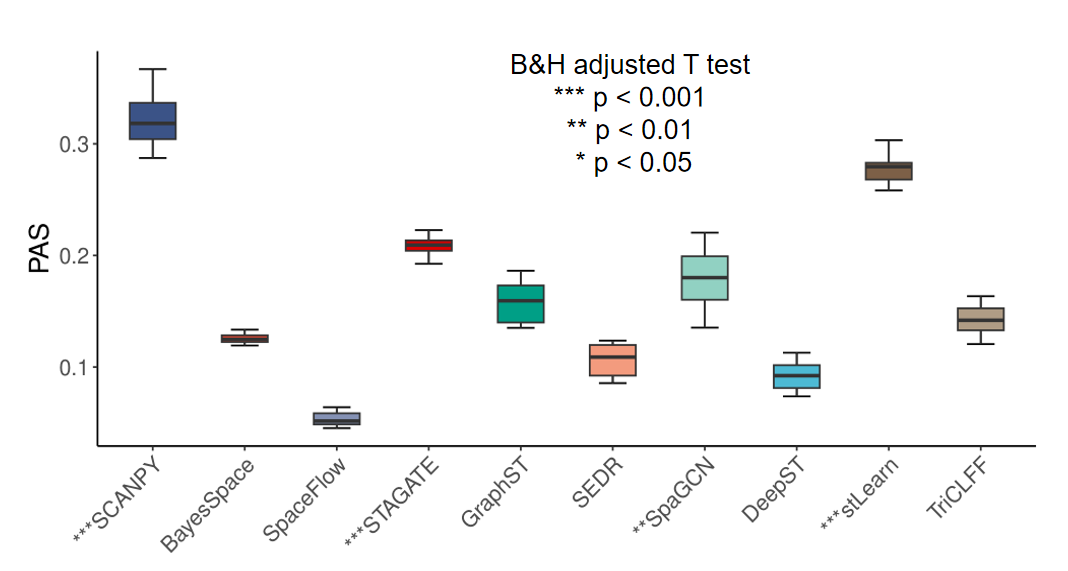
***

**Supplementary Figure S23.** Spatial homogeneity evaluated by PAS on breast cancer data. Stars near the name of method indicate statistical significance of TriCLFF superior to the corresponding method. (one-tailed t-test adjusted by Benjamini-Hochberg method, *:$\boldsymbol{p}<\mathbf{0}.\mathbf{05}$, **: $\boldsymbol{p}<\mathbf{0}.\mathbf{01}$, ***: $\boldsymbol{p}<\mathbf{0}.\mathbf{001}$, no stars: not significant)


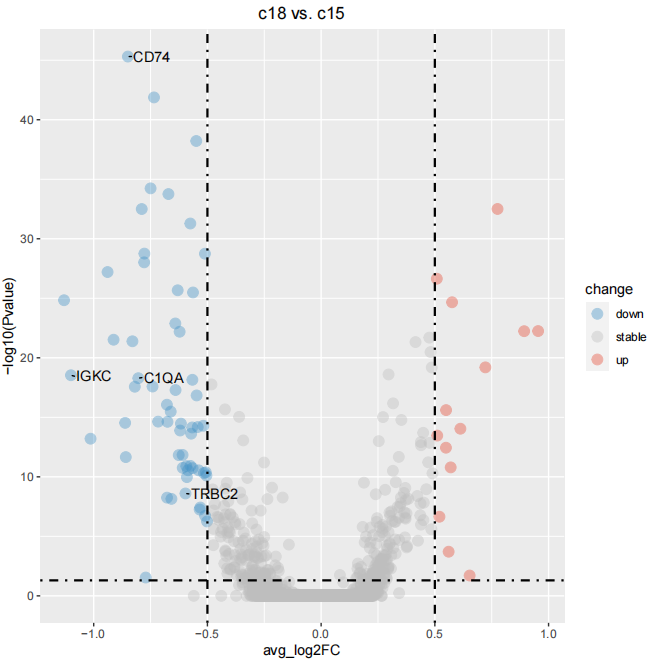


**Supplementary Figure S24.** Volcano graph of differential expression genes (DEGs) between domains 18 and 15. we found 72 DEGs in total.

**
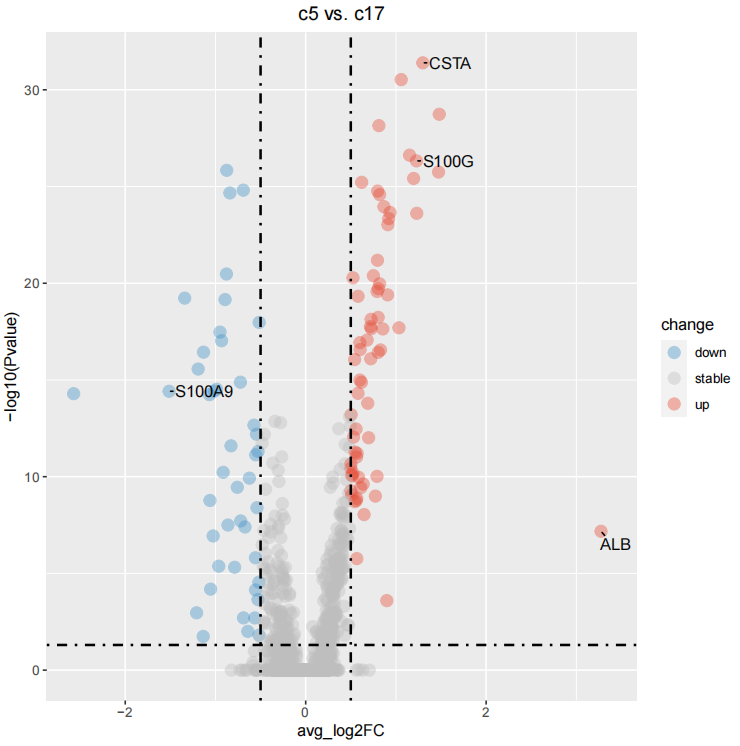
**

**Supplementary Figure S25.** Volcano graph of differential expression genes (DEGs) between regions 5 and 17. We found 111 DEGs in total.


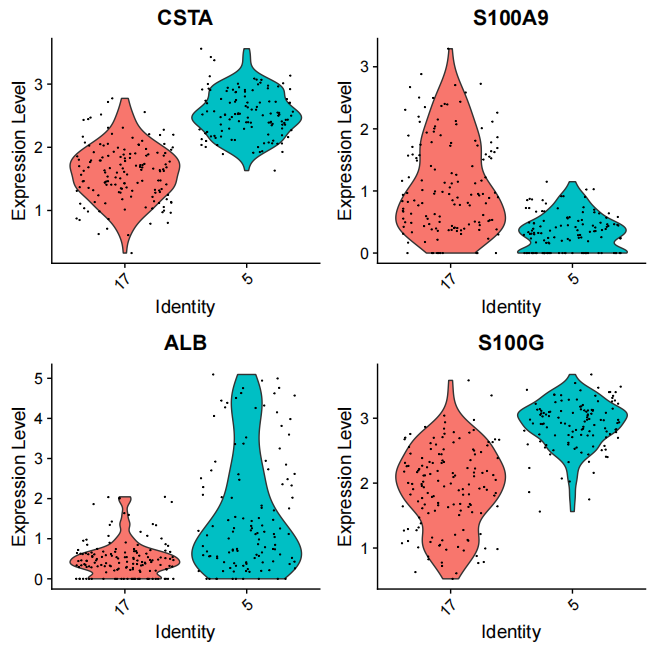


**Supplementary Figure S26.** CSTA, S100A9, ALB, and S100G express differentially between regions 5 and 17.


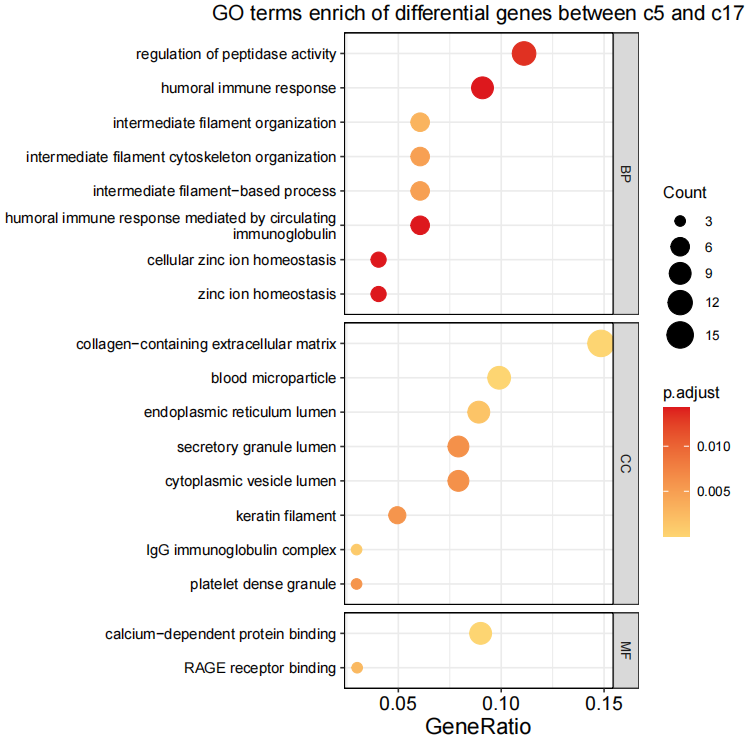


**Supplementary Figure S27.** The Gene Ontology (GO) enrichment analysis of the differential expression genes (DEGs) between regions 5 and 17.

**
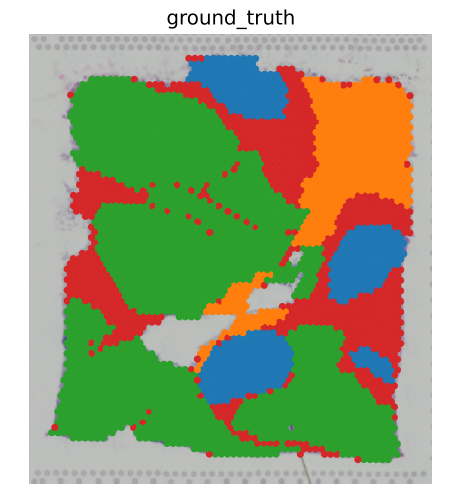
**

**Supplementary Figure S28.** The ground truth maps of breast cancer data classified into four categories according to manual annotation.

**
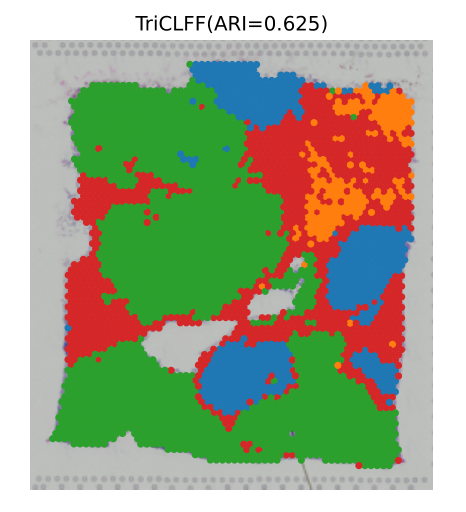
**

**Supplementary Figure S29.** The C1-C5 were further classified into four categories to ensure the consistency between the number of merged regions and the histological type. The ARI score between the merged regions and manual annotations was 0.625, indicating strong alignment between our merged regions and histological annotations.


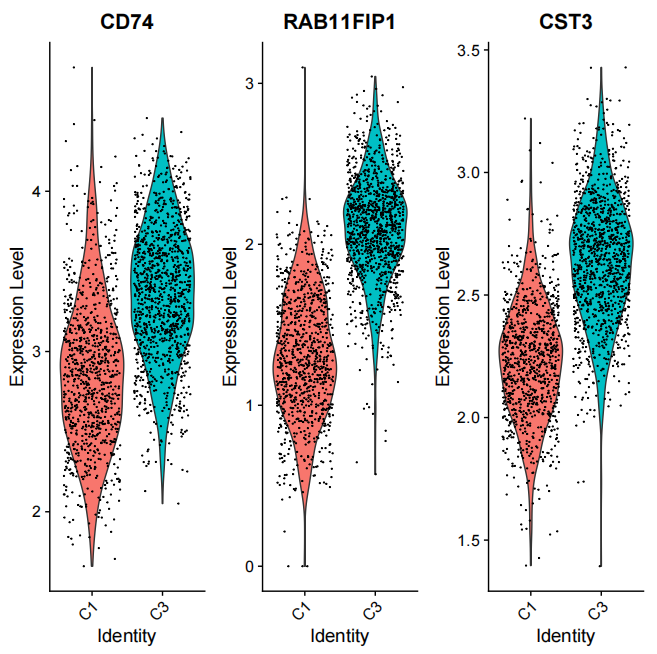


**Supplementary Figure S30.** CD74, RAB11FIP1 and CST3 express differentially between regions C1 and C3.


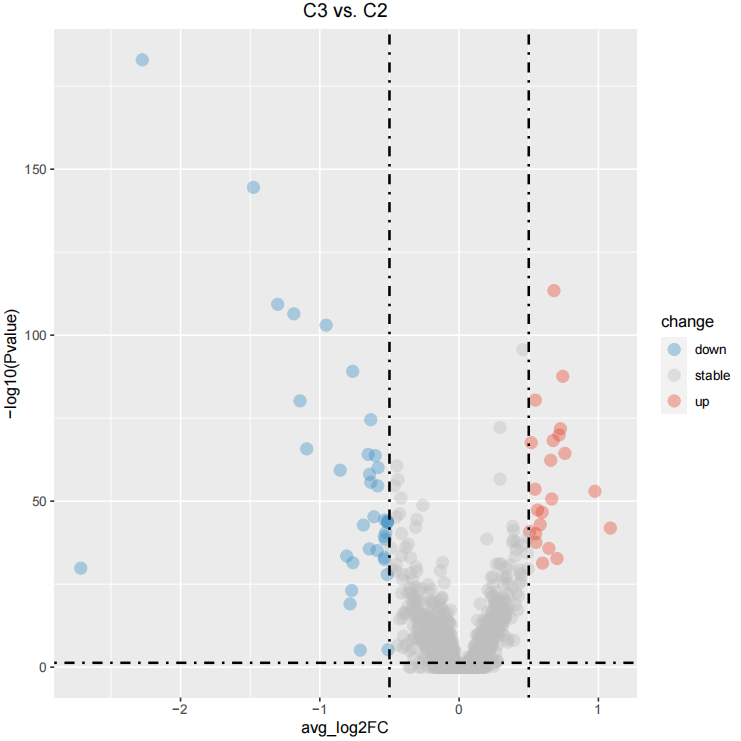


**Supplementary Figure S31.** Volcano graph of differential expression genes (DEGs) between regions C3 and C2.


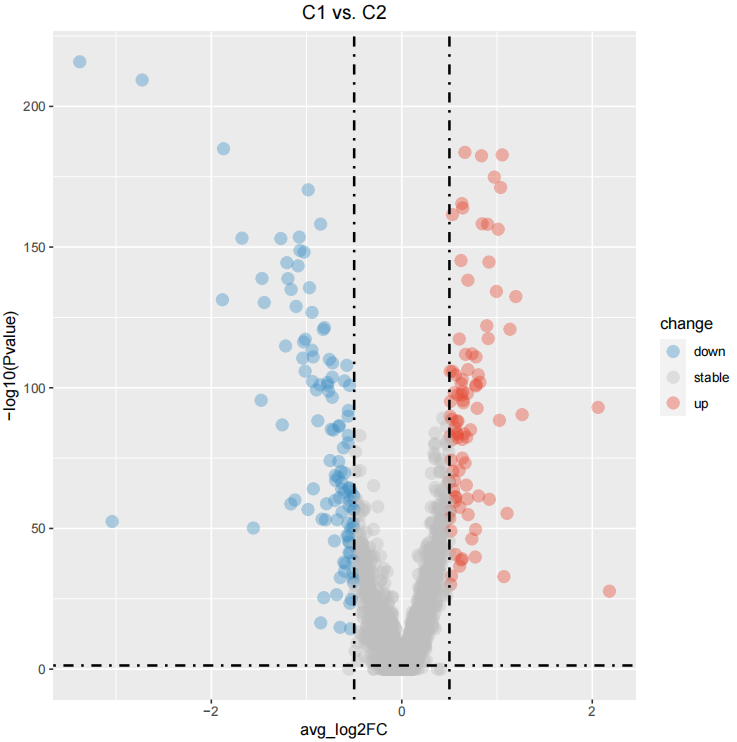


**Supplementary Figure S32.** Volcano graph of differential expression genes (DEGs) between regions C1 and C2.


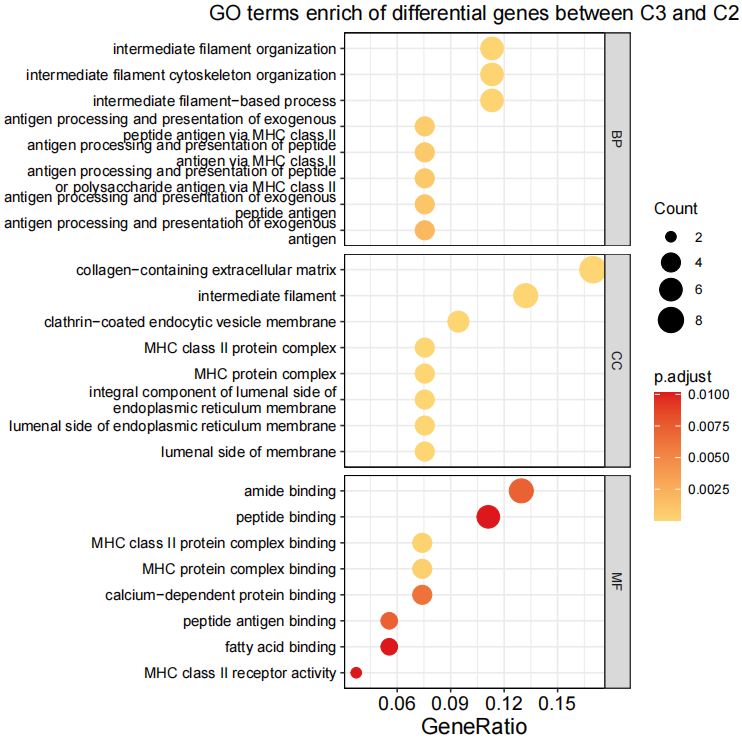


**Supplementary Figure S33.** The Gene Ontology (GO) enrichment analysis of the differential expression genes (DEGs) between regions C2 and C3.


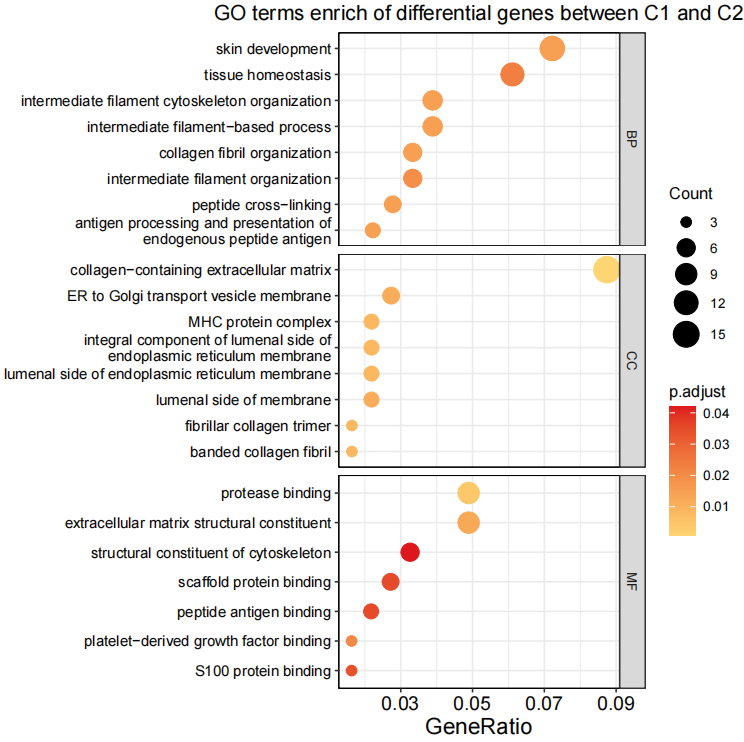


**Supplementary Figure S34.** The Gene Ontology (GO) enrichment analysis of the differential expression genes (DEGs) between regions C1 and C2.
