# Supplementary material for: TriCLFF: a multi-modal feature fusion framework using contrastive learning for spatial domain identification
Source: Brief Bioinform. 2025 Jul 10;26(4):bbaf316. doi: 10.1093/bib/bbaf316 (PMC12245166; doi:10.1093/bib/bbaf316)
Supplement: supplementary-material_bbaf316 [file supplementary-material_bbaf316.zip › Supplementary_Table5_bbaf316.docx]

| **Metrics** | **Formulas** | **Variables in the formulas** |
| --- | --- | --- |
| CHAOS | $CHAOS=\frac{\sum_{k=1}^{K} \sum_{i,j}^{n_{k}} \omega_{kij}}{N}$  $\omega_{kij}=\left\{ \begin{aligned} d_{ij},\mathrm{if} i \mathrm{and} j are connected in the 1NN graph \\ 0, \mathrm{ot}h\mathrm{erwise} \end{aligned} \right.$ | To calculate CHAOS, a one-nearest-neighbor (1NN) graph for the spots in each spatial cluster is created first. $\omega_{kij}$ denotes the edge weight between spot $i$ and spot $j$. $N$ is the total number of spots; $K$ is the total number of spatial domains; and $n_{k}$ is the number of spots in $k$-th spatial domain. $d_{ij}$ is evaluated by Euclidean distance. |
| PAS | $\mathrm{PAS}=\frac{1}{N}\sum_{i=1}^{N} 1\left[ \sum_{j\in\mathcal{N}_{{10}^{\left( i \right)}}} 1\left( y_{j}\neq y_{i} \right)\geq6 \right]$ | The PAS score is calculated as the proportion of spots with a cluster label that is different from at least six of its neighboring ten spots. $N$ represents total number of spots, $y_{i}$ is the cluster label of spot $i$, $\mathcal{N}_{{10}^{\left( i \right)}}$ is the set of 10 nearest neighbors of spot $i$ based on spatial coordinates and 1[⋅] is an indicator function that returns 1 if the condition is true, else 0. |
| NMI | $\mathrm{NMI}\left( U, V \right)=\frac{\mathrm{MI}\left( U,V \right)}{\left[ H\left( U \right)+H\left( V \right) \right]/2}$ | NMI measures the similarity between the predicted labels $U$ and ground truth labels $V$. $MI\left( U,V \right)$ is mutual information between labels $U$ and $V$. $H\left( U \right)$ and $H\left( V \right)$ respectively represent the information entropy of labels $U$ and $V$. |
| AMI | $\mathrm{AMI}\left( U, V \right)=\frac{\left[ \mathrm{MI}\left( U,V \right)-E\left( \mathrm{MI}\left( U,V \right) \right) \right]}{\left[ \mathrm{avg}\left( H\left( U \right),H\left( V \right) \right)-E\left( \mathrm{MI}\left( U,V \right) \right) \right]}$ | $MI\left( U,V \right)$, $H\left( U \right)$, $H\left( V \right)$ are defined as in NMI above. $E\left( MI\left( U,V \right) \right)$ is expected mutual information under random assignment. |
| HOM | $\mathrm{HOM}\left( U, V \right)=1-\frac{H\left( U\vert V \right)}{H\left( U \right)}$ | Homogeneity assesses whether each predicted cluster contains only members of a single class. $H\left( U\vert V \right)$ is the conditional entropy of predicted labels $U$ when given true labels $V$. $H\left( U \right)$ represents the entropy of predicted labels. |
| ICC | $\mathrm{ICC}_{i}^{(k)}=\frac{\sigma_{m}^{2}}{\sigma_{i}^{2}+\sigma_{m}^{2}}$ | For ICC, we used the gene expression modality (top 50 principal components) to calculate both the within-cluster variance ($\sigma_{i}^{2}$) and the variance across cluster means ($\sigma_{m}^{2}$) for each cluster, $i$ is a given cluster, $k$ represents the modality. |

**Supplementary Table 5: The definitions of the supplemented metrics CHAOS, PAS, NMI, AMI, HOM and ICC.**
