# Supplementary material for: TriCLFF: a multi-modal feature fusion framework using contrastive learning for spatial domain identification
Source: Brief Bioinform. 2025 Jul 10;26(4):bbaf316. doi: 10.1093/bib/bbaf316 (PMC12245166; doi:10.1093/bib/bbaf316)
Supplement: supplementary-material_bbaf316 [file supplementary-material_bbaf316.zip › Response_Letter_bbaf316.docx]

**Response to Reviewer 1**

Comments to the Author: The authors have addressed my concerns.

**R: Thanks for your helpful comments during the earlier rounds, which contributed significantly to improving the manuscript. There is nothing in the comments that needs to be responded.**

**Response to Reviewer 2**

Comments to the Author: Accepted.

**R: We are truly grateful for your help with improving the manuscript. There is nothing in the comments that needs to be responded.**

**Response to Reviewer 3**

Most of the issues are well addressed, but some minor points remain. The manuscript is now much clearer and more comprehensive, but it still requires some minor adjustments to enhance clarity and consistency. I recommend accepting the manuscript after minor revisions.

**R: Thanks for your thoughtful and constructive feedback. We are glad to hear that the manuscript is now much clearer and more comprehensive. Following your comments, we have carefully revised the manuscript to further improve its clarity and consistency. All minor points have been addressed in the revised version. We are grateful for your guidance throughout the review process.**

**Minor points:**

1. For the scalability of TriCLFF (Q1(a)), the authors have provided a benchmark on the memoryand time of running for assessing scalability in the revised manuscript. The time usage is significantly higher than other methods and the memory usage is only less than STAGATE, but both of their scales are acceptable.

**R: Thank you for acknowledging our scalability benchmark and the revisions we made in response to Q1(a). We appreciate your assessment that both the time and memory usage are within acceptable ranges. We are grateful for your understanding and supportive evaluation. Since your expression in the comment is much closer to the actual situation, we have made the following adjustment on the narration of scalability (Lines 348-350):**

***“We have provided a benchmark on the memory and time of running for assessing scalability. The time usage is higher than other methods and the memory usage is only less than STAGATE, but both of their scales are acceptable.”***

2. For the additional recommended module of Decoder in MLP (Q1(b)), the authors’ results show that the performance of TriCLFF with Decoder is slightly worse than that without Decoder (seems statistically significant). However, it would be more clear if the used data and other configurations of the ablation experiments, including w/o decoder, searching λ’s, and searching (α, β), are consistent with those of modality fusion experiments (Supplementary Figure S13), or vice versa.

**R：Thank you for your valuable comments. We confirm that all the ablation experiments, including w/ or w/o decoder, searching** **λ’s, and searching (α, β), are performed on the mouse brain dataset in Figure 2. Except for the parameters that need to be tuned, all the parameters are configured as shown in Supplementary Table 4. As modality fusion is a key focus of our work, we further conducted ablation experiments on the section 151672 of human dorsolateral prefrontal (DLPFC) data (Supplementary Figures S11 andS13) and the breast cancer data (Supplementary Figure S12) where the parameters are also set consistent with Supplementary Table 4. We have made minor revisions on the main text to make this point clear (Lines 139-142, 242-243 and 253-254):**

***“The implement details of TriCLFF are described in the following sections while the parameters in main analysis pipelines and ablation experiments (except for the parameters are tunning) are provided in Supplementary Table S4.”***

***“In our analysis, the parameters*** $\lambda_{1}\boldsymbol{\sim}\lambda_{6}$ ***are all set to 0.1 according to the local grid searching on the mouse brain dataset.”***

***“… which are set to*** $\alpha=0.5,\beta=0.1$ ***in our analysis according to grid searching on the mouse brain dataset.”***

3. For evaluating regional continuity and noise (Q4), the authors have added the CHAOS and Percentage of Abnormal Spots (PAS) metrics in the revised manuscript. The results show that TriCLFF achieves Top 3 out of 10 in CHAOS and Top 6 out of 10 in PAS, so the authors revised the claim to “In addition, spatial domains identified by TriCLFF has approximate or better regional continuity”, which is rigorous and acceptable.

**R：Thank you very much for your careful review and positive feedback. We are glad that the inclusion of the CHAOS and PAS metrics, as well as the adjustment of our claim, has addressed your concern appropriately.**

4. For repository (Q5), still the code is hard to run due to the issues in environment configuration, such as the version of torchtoolbox (not listed in requirements.txt) and STAGATE.pyG==1.0.0 should be excluded from requirements.txt since it can not be installed by pip install directly. It is highly recommended to try it by a third person and debug the issues before release.

**R：Thank you for pointing out the issues regarding the reproducibility of the code and environment configuration. In response, we have carefully revised the requirements.txt file to include the correct version of torchtoolbox, and we have removed STAGATE.pyG==1.0.0 since our code doesn't need the STAGATE package. Furthermore, we asked a third person to independently run and test the code following the updated instructions. Necessary adjustments and clarifications have been made to ensure a smoother setup experience. We truly appreciate your recommendation, which significantly helped us improve the usability and accessibility of our repository.**

5. For evaluating spatial clustering accuracy and quality (Q6), the authors have added the benchmarks on NMI, AMI, HOM, and ICC metrics in the revised manuscript. The results shows that TriCLFF consistently achieves Top 1 out of 10 in NMI, AMI, and HOM, but Top 4 out 10 in ICC, which is acceptable.

**R：Thank you for your approval. We appreciate your previous valuable comments that helped us with improving assessment strategies.**

6. For the added metrics, i.e. CHAOS, PAS, NMI, AMI, HOM, and ICC (Q4 and Q6), formulas should be provided in the main text or supplementary materials for clarity. For example, in $\text{ICC}_{i}=\frac{\sigma_{m}^{2}}{\sigma_{i}^{2}+\sigma_{m}^{2}}$, which modality did you use to calculate $\sigma_{i}^{2}$ and $\sigma_{m}^{2}$, and how to summarise the ICC values of all clusters? The same question also exists in the metrics involving distance calculations, such as CHAOS.

**R：Thank you for your careful examination and valuable comments regarding the clarity of the metrics definitions. Due to the length of the whole manuscript, we have added the definitions of CHAOS, PAS, NMI, AMI, HOM, and ICC to the Supplementary Materials (Supplementary Table 5).**

**Specifically, for ICC, we used the gene expression modality (top 50 principal components) to calculate both the within-cluster variance (**$\boldsymbol{\sigma}_{\boldsymbol{i}}^{\mathbf{2}}$**) and the variance across cluster means (**$\boldsymbol{\sigma}_{\boldsymbol{m}}^{\mathbf{2}}$**) for each cluster. The overall ICC for a method is the average of the ICCs across all clusters. For CHAOS, the one-nearest-neighbor graph for each predicted cluster is constructed based on Euclidean distance between spots within the cluster. The PAS score is calculated as the proportion of spots with a cluster label that is different from at least six of its neighboring ten spots. For NMI, AMI, and HOM, we followed the standard definitions used in clustering evaluation. To better understand the usage of these metrics, we have made the following adjustment in the main text (Lines 288-295).**

***“Meanwhile, Homogeneity (HOM) and average intraclass correlation coefficient (ICC) on top fifty principal components are used to evaluate the clustering quality.***

***In particular, we used CHAOS on Euclidean distance to assess spatial continuity where lower values indicate better. Meanwhile, PAS (i.e., the proportion of spots with a cluster label that is different from at least six of its ten neighbors) is used to evaluate the homogeneity of spatial domains. For clarity, we provide the definition of the supplemented metrics (i.e. CHAOS, PAS, NMI, AMI, HOM, and ICC) in Supplementary Table S5.”***

***Supplementary Table 5: The definitions of the supplemented metrics CHAOS, PAS, NMI, AMI, HOM and ICC.***

| **Metrics** | **Formulas** | **Variables in the formulas** |
| --- | --- | --- |
| **CHAOS** | $CHAOS=\frac{\sum_{k=1}^{K} \sum_{i,j}^{n_{k}} \omega_{kij}}{N}$  $\omega_{kij}=\left\{ \begin{aligned} d_{ij},if i and j are connected in the 1NN graph \\ 0,otherwise \end{aligned} \right.$ | **To calculate CHAOS, a one-nearest-neighbor (1NN) graph for the spots in each spatial cluster is created first.** $\omega_{kij}$ **denotes the edge weight between spot** $i$ **and spot** $j$**.** $N$ **is the total number of spots;** $K$ **is the total number of spatial domains; and** $n_{k}$ **is the number of spots in** $k$**-th spatial domain.** $d_{ij}$ **is evaluated by Euclidean distance.** |
| **PAS** | $PAS=\frac{1}{N}\sum_{i=1}^{N} 1\left[ \sum_{j\in\mathcal{N}_{{10}^{\left( i \right)}}} 1\left( y_{j}\neq y_{i} \right)\geq6 \right]$ | **The PAS score is calculated as the proportion of spots with a cluster label that is different from at least six of its neighboring ten spots.** $\boldsymbol{N}$ **represents total number of spots,** $y_{i}$ **is the cluster label of spot** $i$**,** $\mathcal{N}_{{10}^{\left( i \right)}}$ **is the set of 10 nearest neighbors of spot** $i$ **based on spatial coordinates and 1[⋅] is an indicator function that returns 1 if the condition is true, else 0.** |
| **NMI** | $NMI\left( U, V \right)=\frac{MI\left( U,V \right)}{\left[ H\left( U \right)+H\left( V \right) \right]/2}$ | **NMI measures the similarity between the predicted labels** $U$ **and ground truth labels** $V$**.** $MI\left( U,V \right)$ **is mutual information between labels** $U$ **and** $V$**.** $H\left( U \right)$ **and** $H\left( V \right)$ **respectively represent the information entropy of labels** $U$ **and** $V$**.** |
| **AMI** | $AMI\left( U, V \right)=\frac{\left[ MI\left( U,V \right)-E\left( MI\left( U,V \right) \right) \right]}{\left[ avg\left( H\left( U \right),H\left( V \right) \right)-E\left( MI\left( U,V \right) \right) \right]}$ | $MI\left( U,V \right)$, $H\left( U \right)$, $H\left( V \right)$ **are defined as in NMI above.** $E\left( MI\left( U,V \right) \right)$ **is expected mutual information under random assignment.** |
| **HOM** | $HOM\left( U, V \right)=1-\frac{H\left( U\vert V \right)}{H\left( U \right)}$ | **Homogeneity assesses whether each predicted cluster contains only members of a single class.** $H\left( U\vert V \right)$ **is the conditional entropy of predicted labels** $U$ **when given true labels** $V$**.** $H\left( U \right)$ **represents the entropy of predicted labels.** |
| **ICC** | $\mathrm{ICC}_{i}^{(k)}=\frac{\sigma_{m}^{2}}{\sigma_{i}^{2}+\sigma_{m}^{2}}$ | **For ICC, we used the gene expression modality (top 50 principal components) to calculate both the within-cluster variance (**$\sigma_{i}^{2}$**) and the variance across cluster means (**$\sigma_{m}^{2}$**) for each cluster,** $i$ **is a given cluster,** $k$ **represents the modality.** |

7. Formula of ARI in Line 270 is still incorrect (Q7).

**R：Thank you for your valuable comments and pointing out this careless error. We have modified the numerator part of the equation. The revised formula is shown as follows (Line 271):**

$$ARI=\frac{\sum_{ij} \left( \begin{matrix} n_{ij} \\ 2 \end{matrix} \right)-\left[ \sum_{i} \left( \begin{matrix} a_{i} \\ 2 \end{matrix} \right)\sum_{j} \left( \begin{matrix} b_{j} \\ 2 \end{matrix} \right) \right]/\left( \begin{matrix} n \\ 2 \end{matrix} \right)}{\frac{1}{2}\left[ \sum_{i} \left( \begin{matrix} a_{i} \\ 2 \end{matrix} \right)+\sum_{j} \left( \begin{matrix} b_{j} \\ 2 \end{matrix} \right) \right]-\left[ \sum_{i} \left( \begin{matrix} a_{i} \\ 2 \end{matrix} \right)\sum_{j} \left( \begin{matrix} b_{j} \\ 2 \end{matrix} \right) \right]/\left( \begin{matrix} n \\ 2 \end{matrix} \right)}$$

8. In Supplementary Figure S7-S8, the rules of marking the significant levels are different with the rest of the figures. Please make them consistent.

**R：Thank you very much for pointing out the marking issue of the significance levels in Supplementary Figures S7–S8. We apologize for error marking of statistic testing methods since Figures S7 and S8 are tested by one-sided *F*-test on variations for the stability under noise. We set the significant level** $\boldsymbol{p<0.1}$ **in this experiment due to the limited repeats while other figures are tested under *t*-test at** $\boldsymbol{p<0.05}$ **(Lines 297). To maintain consistency and clarity, we have adjusted the significance marks in Supplementary Figures S7 and S8 accordingly. In the revised figures, significant levels** $\boldsymbol{p<0.05, 0.01}$ **and** $\boldsymbol{0.001}$ **are marked as *, **, and *** to keep consistent with other boxplots while** $\boldsymbol{p<0.1}$ **is marked as #.**

9. The symbols of projected embeddings in formula (7) in Line 225 are still missing ⋅ ̃

**R：****Thank you for your valuable comments. We feel sorry for this careless error. We have ensured the “~” has been marked in our latest manuscript. The eq. (7) (Lines 226-227) has been corrected to:**

$$\begin{aligned} l_{i,j}=-\log\frac{\exp\left( {s \left( \tilde{z}_{i},\tilde{z}_{j} \right)}/\tau\right)}{\sum_{k=1}^{2N} \mathbb{1}_{\left[ k\neq i \right]}\exp\left( {s \left( \tilde{z}_{i},\tilde{z}_{k} \right)}/\tau\right)} \end{aligned}$$
